# Cloud attenuation estimation based on cloud particle sensor sonde observations for next-generation satellite communications

**DOI:** 10.1038/s41598-026-61143-z

**Published:** 2026-07-08

**Authors:** Takahiro Ohno, Munehiro Matsui, Kiyohiko Itokawa, Tomohiro Tokuyasu, Kosuke Ito, Shoichi Shige, Tetsuya Takemi

**Affiliations:** 1 Access Network Service Systems Laboratories, NTT, Inc., Yokosuka, 239-0847 Japan; 2 Graduate School of Science, Kyoto University, Kyoto, 606-8502 Japan; 3 Disaster Prevention Research Institute, Kyoto University, Uji, 611-0011 Japan

**Keywords:** Cloud attenuation, CPS sonde, Radio frequency, Free space optics, Satellite communication, Meteorological satellite, Climate sciences, Planetary science

## Abstract

Cloud attenuation due to cloud particles can be a critical issue for next-generation sub-terahertz (sub-THz), terahertz (THz), and free-space optical (FSO) satellite communications. Accurate estimation of cloud attenuation under various cloud conditions is essential for the design of satellite communication systems; accordingly, numerous cloud attenuation estimation models have been proposed. The widely used ITU-R model proposed up to 200 GHz is based on liquid water content and does not explicitly consider ice crystals. Previous studies have often assumed water droplets to be dominant, potentially leading to unrealistic cloud attenuation estimates, especially in multilayered cloud systems where ice crystals can exist over a wide temperature range. In addition, approaches based on reanalysis data and numerical weather prediction models rely on parameterized cloud representations and may involve substantial uncertainties. These limitations highlight the need for observationally grounded evaluations that explicitly account for both water droplets and ice crystals. In this study, cloud attenuation was estimated up to wavelengths of approximately 20 $\mu \textrm{m}$ (corresponding to about 10 THz) using ten cloud microphysical data obtained from cloud particle sensor (CPS) sondes observed in Okinawa, Japan, during the Baiu (East Asia rainy season) in 2016, 2017, and 2025. CPS sondes enable the vertical measurement of the number of cloud particles along the sonde flight path and allow for their rough classification into water droplets and ice crystals, making them particularly well-suited to addressing the aforementioned challenges. Cloud attenuation was estimated based on in situ observational data obtained using CPS sondes. To the best of our knowledge, this study is the first to estimate cloud attenuation for satellite communications using in situ observational data of cloud particles. The results demonstrate that cloud attenuation due to ice crystals becomes dominant, particularly at wavelengths shorter than 1.0 mm, challenging the conventional picture that cloud attenuation is dominated by water droplets. This finding was obtained by explicitly considering the size distribution of ice crystals. A key contribution of this study is the identification of limitations in conventional cloud attenuation models for satellite communications using in situ cloud particle observations. Expanding similar studies globally could lead to the development of more accurate and widely applicable cloud attenuation models.

## Introduction

In the field of satellite communications, evaluating the interaction between electromagnetic waves and clouds is important. Next-generation mobile communication systems, such as Beyond 5G and 6G, aim to provide ultra-wide coverage, thereby enabling mobile services in underserved and remote areas. In this context, satellite communications have recently attracted increasing attention as a key enabling technology. Conventional satellite communications have primarily been used for specialized applications, such as disaster response and telephony relay, where the communication demand has been relatively limited. Therefore, systems operating in the Ka- and Ku-bands have been sufficient to meet these requirements. However, next-generation satellite communications, expected as part of mobile networks, must accommodate rapidly increasing demand. Beyond the conventional Ka- and Ku-bands, there is growing interest in higher-frequency technologies for high-speed and high-capacity communications, including sub-terahertz (sub-THz), terahertz (THz), and free-space optical (FSO) systems, corresponding to wavelength ranges of 3 mm–1 mm (100 GHz–300 GHz), 1 mm–$30~\mu \textrm{m}$ (300 GHz–10 THz), and below $30~\mu \textrm{m}$ (above 10 THz, including long-wave infrared FSO systems), respectively^1–4^. These sub-THz, THz, and FSO wavelength ranges are all under consideration, suggesting that next-generation satellite communications will depend on a wide range of wavelengths rather than a single spectral band.

In satellite communications based on sub-THz, THz, and FSO, the presence of clouds along the propagation path can cause significant signal attenuation, hereafter referred to as cloud attenuation, due to absorption and scattering by cloud particles through Mie scattering processes. Therefore, to achieve stable communication quality and highly reliable networks, it is essential to quantitatively evaluate the relationship between cloud physical parameters and cloud attenuation. Unlike inter-satellite communication links, which operate under near-vacuum conditions, ground-to-satellite communication links are affected by clouds and atmospheric conditions. Atmospheric and cloud effects are among the primary factors limiting the availability of sub-THz, THz, and FSO systems, as they can cause severe signal attenuation and even complete communication outages^5,6^. Therefore, accurate characterization of cloud attenuation is essential for reliable channel modeling, link design, and bit-error rate performance evaluation in satellite communications based on sub-THz, THz, and FSO systems. Previous studies on free-space and satellite optical communications, including advanced techniques such as orbital angular momentum-based systems, have highlighted the importance of accurate channel modeling under atmospheric impairments^7^. As discussed later, this study provides a valuable contribution to this need by addressing the limitations of conventional cloud attenuation models. Beyond communications, meteorological observation satellites, particularly cloud-observing missions such as EarthCARE, employ millimeter-wavelength radio waves^8^. Understanding how electromagnetic waves at these wavelengths interact with clouds is essential for the calibration and interpretation of these observational data. Meteorological observation satellites utilize the electromagnetic waves reflected by target objects after transmission^9,10^. Although the scattering direction of interest differs from that in satellite communications, the underlying principles are common.

A widely used and standardized approach for estimating cloud attenuation in the field of satellite communications is provided by Recommendation P.840-9 of the International Telecommunication Union Radiocommunication Sector (ITU-R), hereafter referred to as the ITU-R model^11^. The ITU-R model provides a method for estimating cloud attenuation for radio waves in the frequency range of 1 to 200 GHz. The ITU-R model does not extend to the THz and FSO regimes but can still be used to evaluate cloud attenuation in the sub-THz range. In this model, the cloud liquid water content (LWC), defined as the mass of liquid water per unit volume, or the cloud liquid water path (LWP), defined as the vertical integral of LWC per unit area, is used as an input parameter. It should be noted that LWC and LWP are parameters defined for liquid water. In this framework, the contribution of ice crystals is not explicitly discussed. However, in principle, water droplets and ice crystals should be distinguished because their physical properties in the scattering process differ significantly. In previous studies on cloud attenuation in satellite communications, attenuation due to water droplets has generally been regarded as dominant, while attenuation due to ice crystals has often been considered negligible^12,13^. In some cases, cloud attenuation attributable to ice crystals has even been treated as a constant and discussed accordingly, rather than being addressed with the same level of rigor as cloud attenuation due to water droplets^14,15^. In these previous studies, the ice clouds considered were limited to relatively thin cirrus clouds with thicknesses on the order of 1 km to several kilometers, and clouds at lower altitudes than cirrus were not treated as ice clouds. Clouds often exhibit a multilayered structure. Cirrus clouds, which are typically located in the uppermost layer, exist in environments where temperatures fall below $-40^\circ \textrm{C}$, and their cloud particles therefore occur in the form of ice crystals^16^. On the other hand, in the temperature range between $0^\circ \textrm{C}$ and $-40^\circ \textrm{C}$, ice crystals and supercooled water droplets can coexist^17^. Therefore, it is reasonable to assume that middle clouds located below cirrus may also contain ice crystals. Based on the above considerations, it is possible that the impact of ice crystals on cloud attenuation has been underestimated in previous studies in the field of satellite communications.

Several approaches to evaluating cloud attenuation using reanalysis data and numerical weather prediction models have also been investigated. One study estimates cloud attenuation using Mie scattering theory by prescribing particle size distributions (PSDs) for different cloud types^18^, while another estimates cloud attenuation by applying Mie scattering theory to LWC derived from reanalysis meteorological databases^19^. Some studies have attempted to model the three-dimensional spatial distribution of clouds using numerical weather prediction products, with the aim of incorporating these three-dimensional cloud structures into cloud attenuation estimation models^20,21^. These approaches rely on idealized or parameterized representations of clouds rather than on in situ observations of cloud particles, and they perform theoretical calculations based on these modeled cloud properties. However, it has been reported that values provided by reanalysis data and numerical weather prediction models can differ by orders of magnitude from those obtained through observations, for example in the case of LWC and ice-nucleating particles^22–24^. This suggests that the values derived from reanalysis data and numerical weather prediction models inherently involve uncertainties and ambiguities. Therefore, investigations that depend heavily on reanalysis data and numerical weather prediction models may be subject to substantial risks and limitations.

In this study, we address the aforementioned issues by estimating cloud attenuation using cloud microphysical data obtained through in situ observations. In general, methods for observing cloud microphysical data include aircraft observations, radar observations, and radiosonde observations. Aircraft observations are generally performed during horizontal flight, enabling the acquisition of data for a particular cloud layer. Radar observations generally detect raindrops with diameters ranging from approximately 0.1 mm to several millimeters. Detecting cloud particles with diameters on the order of several tens of micrometers requires specialized radar systems, which are currently very limited in operational use in Japan. Because satellite communication signals propagate between the ground and space, an observational method capable of easily resolving the vertical structure of cloud layers is desirable. However, aircraft observations are typically limited to measurements within specific cloud layers, and radar systems capable of detecting cloud particles are rarely available. In radiosonde observations, the use of cloud particle sensor (CPS) sondes, as described in Fujiwara et al. (2016), enables the vertical counting of cloud particles along the sonde flight path and allows them to be roughly classified into water droplets and ice crystals^25^. Therefore, CPS sondes are well suited for the observations conducted in this study. As the location and period for the CPS sonde observations, we selected Okinawa, Japan during the Baiu (East Asia rainy season) for the following reasons. During the Baiu, a variety of cloud systems develop in an organized manner along the Baiu front, ranging from mesoscale to synoptic scale^26^. Because Okinawa is surrounded by the ocean on all sides, it is a region where CPS sonde observations can be conducted relatively easily in terms of operations. Based on the above considerations, we analyzed ten CPS sondes in Okinawa during the Baiu of 2016, 2017, and 2025. The use of multiple in situ observational datasets reduces uncertainties associated with complex physical assumptions and retrieval processes. Furthermore, unlike satellite observations except for sounders, which provide information only at specific wavelengths, in situ cloud particle measurements allow investigation across a wide range of wavelengths. Cloud attenuation is estimated using a model based on the established framework of Mie scattering theory and the radiative transfer equation, in which both water droplets and ice crystals are explicitly considered, with in situ observational data incorporated into the analysis. In this study, we focus on the sub-THz, THz, and FSO wavelength ranges in which Mie scattering theory is applicable, specifically down to wavelengths of approximately $20~\mu \textrm{m}$ (corresponding to about 10 THz). The objective of this study is to provide a fundamental understanding of how the meteorological and environmental conditions of clouds affect satellite communications through the analysis of cloud attenuation. A key contribution of this study is the estimation of cloud attenuation based on in situ cloud particle observations, which highlights the limitations of the ITU-R model below 200 GHz and the importance of ice crystals in cloud attenuation. In addition, cloud attenuation was evaluated down to wavelengths of approximately $20~\mu \textrm{m}$ (corresponding to about 10 THz) by incorporating the effects of ice crystals that are not considered in conventional models.

The rest of this paper is organized as follows. The Methods section provides a detailed description of the cloud attenuation estimation model used in this study, as well as the cloud particle data observed with CPS sondes. The Results section presents the estimated cloud attenuation categorized by weather condition and summarizes their overall trends and characteristics. The Discussion section examines the influence of ice crystals on cloud attenuation and the differences in cloud attenuation associated with variations in the weather condition.

## Methods

### Cloud attenuation estimation method

#### Conceptual framework

Cloud attenuation of electromagnetic waves arises from the cumulative effect of Mie scattering by individual cloud particles. In the regions where the wavelength is sufficiently larger than the size of cloud particles, scattering is often treated using Rayleigh theory rather than Mie theory. However, Rayleigh scattering can be regarded as a limiting case of Mie scattering. Therefore, in this study, we investigate wavelength ranges in which Mie scattering theory is applicable, including the conventional Ka- and Ku-band regions, and perform analyses over wavelengths from 3 cm to approximately $20~\mu \textrm{m}$ (corresponding to frequencies from 10 GHz to about 10 THz). In this study, cloud attenuation was estimated by first calculating the scattering parameters of a single cloud particle based on Mie scattering theory, and then evaluating the cumulative effect by linearly accumulating the scattering contributions from individual cloud particles using the radiative transfer equation. For computational simplicity, we focused on the fraction of all photons incident on the cloud that pass through it without undergoing any absorption or scattering by cloud particles. Accordingly, only single-scattering processes by cloud particles were considered, and multiple scattering effects were not considered. This assumption has been reported to be reasonably valid for FSO, as scattered photons are rarely detected by the receiver^18^. Furthermore, when this assumption is extended to backscattering and applied to the evaluation of radar reflectivity in meteorological satellites and weather radars, it is useful for mitigating and correcting biases in reflected signals caused by multiple scattering^27^.

Here, we consider the most fundamental case of Mie scattering by a spherical scatterer, in which a single scatterer is located at the origin of an arbitrary Cartesian coordinate system and a linearly polarized electromagnetic wave propagates toward the scatterer. In this configuration, the extinction cross section, $\sigma _\mathrm{{ext}}$, resulting from Mie scattering is expressed as a function of the complex refractive index and the size of the scatterer. Cloud attenuation was determined by applying $\sigma _\mathrm{{ext}}$ to the radiative transfer equation.

The radiative transfer equation models electromagnetic wave intensity variation due to absorption and emission in a medium. The infinitesimal change *dI* in the intensity *I* of an electromagnetic wave after traveling a distance *ds* in a medium with absorption coefficient *κ* and emissivity *ε* is expressed as

1 $$\begin{aligned} dI = \epsilon ds-\kappa Ids. \end{aligned}$$

In this study, we primarily focus on cloud attenuation of signals in satellite communications. Since electromagnetic waves absorbed by cloud particles are re-emitted without retaining the information of the original signal, the re-emitted component reaching the receiver is excluded from the analysis. For this reason, we consider only the absorption term in the radiative transfer equation and neglect the emission term. Furthermore, by defining the optical depth *τ* as $d\tau \equiv \kappa ds$ and solving the differential equation (1), we obtain

2 $$\begin{aligned} \frac{I}{I_0} = e^{-\tau }, \end{aligned}$$

where $I_0$ represents the intensity of the incident electromagnetic wave at the position corresponding to *ds=0*. Equation (2) represents the ratio of the intensity of the electromagnetic wave after propagating through a medium to that of the incident electromagnetic wave, and therefore directly quantifies the attenuation induced by the medium.

Based on the definition, the optical depth *τ* is given by the product of the extinction cross section $\sigma _\mathrm{{ext}}$, the cloud particle number density $N_{cp}$, and the cloud layer thickness $L_c$.

3 $$\begin{aligned} \tau = \sigma _\mathrm{{ext}}\times N_{cp}\times L_c. \end{aligned}$$

Here, considering that CPS sondes are capable of resolving the vertical structure of cloud layers, we replace $L_c$ with the position of the CPS sonde, denoted by *x*. When the CPS sonde moves from position *x* to *x+Δ x* at a given observation time, let the mean extinction cross section of the observed cloud particles be denoted by $\overline{\sigma _{\textrm{ext}}(x,x+\Delta x)}$, and the mean number density by $\overline{N_{cp}(x,x+\Delta x)}$. Here, *Δ x* can be interpreted, for example, as the distance traveled (i.e., ascent) by the CPS sonde during the time interval over which measurements are recorded. The corresponding mean optical depth is then given by

4 $$\begin{aligned} \overline{\tau (x,x+\Delta x)} = \overline{\sigma _{\textrm{ext}}(x,x+\Delta x)} \times \overline{N_{cp}(x,x+\Delta x)} \times \Delta x. \end{aligned}$$

It should be noted that $\overline{N_{cp}(x,x+\Delta x)}=0$ in regions where no cloud particles are present. Thus, the cloud attenuation over the interval from *x* to *x+Δ x* in dB is given by

5 $$\begin{aligned} A_{c}(x,x+\Delta x)=-10\log {\left[ \exp {\left\{ -\overline{\tau (x,x+\Delta x)}\right\} }\right] }. \end{aligned}$$

The cloud attenuation along the vertical direction is given by

6 $$\begin{aligned} A_{c} = \sum _{x} A_{c}(x, x+\Delta x). \end{aligned}$$

In this study, cloud attenuation was evaluated based on equation (6) using cloud particle size and number density as functions of the CPS sonde position, obtained from CPS sonde observations. It should be noted that the cloud attenuation given by equation (6) was calculated separately for water droplets and ice crystals, and the total cloud attenuation was obtained by summing these contributions.

In the calculation of the extinction cross section, water droplets and ice crystals were distinguished based on their complex refractive indices and particle shapes. Water droplets were approximated as spheres, whereas ice crystals were represented by two types of cylindrical particles with different aspect ratios. The scattering parameters for both water droplets and ice crystals were calculated using the T-matrix method^28,29^, which allows scattering by nonspherical particles to be evaluated beyond the assumption of spherical symmetry inherent in classical Mie scattering theory.

The following sections provide a detailed description of the modeling of water droplets and ice crystals, and T-matrix calculation settings. The subsequent section describes the characteristics of the constructed cloud attenuation estimation method and its relationship with the PSD of cloud particles.

#### Modeling of cloud particles and T-matrix calculation settings

In calculating the extinction cross sections of cloud particles, the following approximations were applied to water droplets and ice crystals. For water droplets, a relatively simple assumption was adopted, whereby they were treated as spherical scatterers. The complex refractive index of liquid water varies with wavelength; therefore, wavelength-dependent values reported in the previous study were used^30^. For ice crystals, their characteristics have been reported in previous observational studies as follows. According to HYdrometeor VIdeoSonde (HYVIS) observations conducted in Okinawa during the Baiu, plate-like and columnar ice crystals are the representative crystal habits and occur in approximately equal proportions^31^. Furthermore, aircraft observations have indicated that the aspect ratios of ice crystals are approximately 0.6^32^. Based on these findings, ice crystals in this study were assumed to consist of an equal mixture of plate-like and columnar particles, and both types were approximated as cylindrical scatterers. In the T-matrix calculations, the axis ratio (defined as the horizontal axis to the rotational axis) was used as a shape parameter. The axis ratios of cylindrical particles were set to $0.6^{-1} \approx 1.7$ for plate-like ice crystals and 0.6 for columnar ice crystals, respectively. As with liquid water, the complex refractive index of ice is wavelength dependent; therefore, literature values from the previous study were adopted^33^.

The extinction cross sections of cloud particles were calculated using the T-matrix method implemented in the pytmatrix package, a Python-based computational package. When specifying cloud particle sizes in pytmatrix, the particle sizes observed with CPS sondes were provided as equivalent volume diameters. For particle shape specification, spheroidal geometries were assigned for water droplets, whereas cylindrical geometries were assigned for ice crystals by selecting the corresponding shape identifiers in pytmatrix. In addition, cloud particles were assumed to be randomly oriented, and the zenith angles of both the incident and scattered electromagnetic waves were set to 180$^{\circ }$, corresponding to wave propagation from the upper atmosphere toward the surface.

#### Characteristics of the cloud attenuation estimation method

Before evaluating cloud attenuation in the real world, we describe the relationships between several hypothetical cloud particles and their scattering magnitudes. The hypothetical cloud particles used in this section are nine representative cloud particles as summarized in Table 1. For each case the extinction efficiency factor, $Q_\mathrm{{ext}}$, defined as the extinction cross section normalized by the geometric cross-sectional area of a single cloud particle, was calculated using the T-matrix method.

**Fig. 1:**
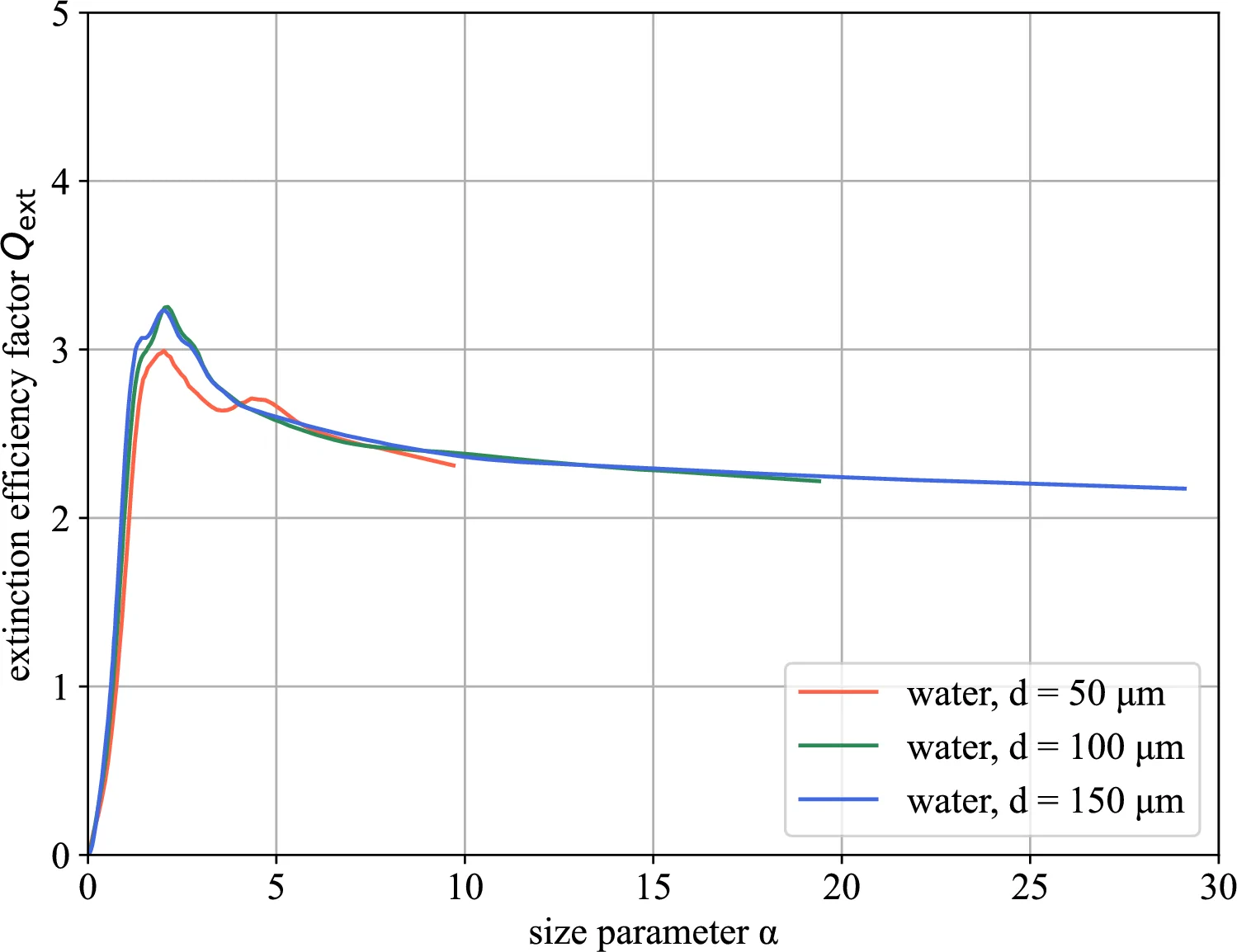
Extinction efficiency factors computed using the cloud attenuation estimation method for water droplets with three different diameters. The horizontal axis represents the size parameter, $\alpha = \pi d_\mathrm{{particle}} / \lambda$, and the vertical axis represents the extinction efficiency, $Q_\mathrm{{ext}}$.

**Fig. 2:**
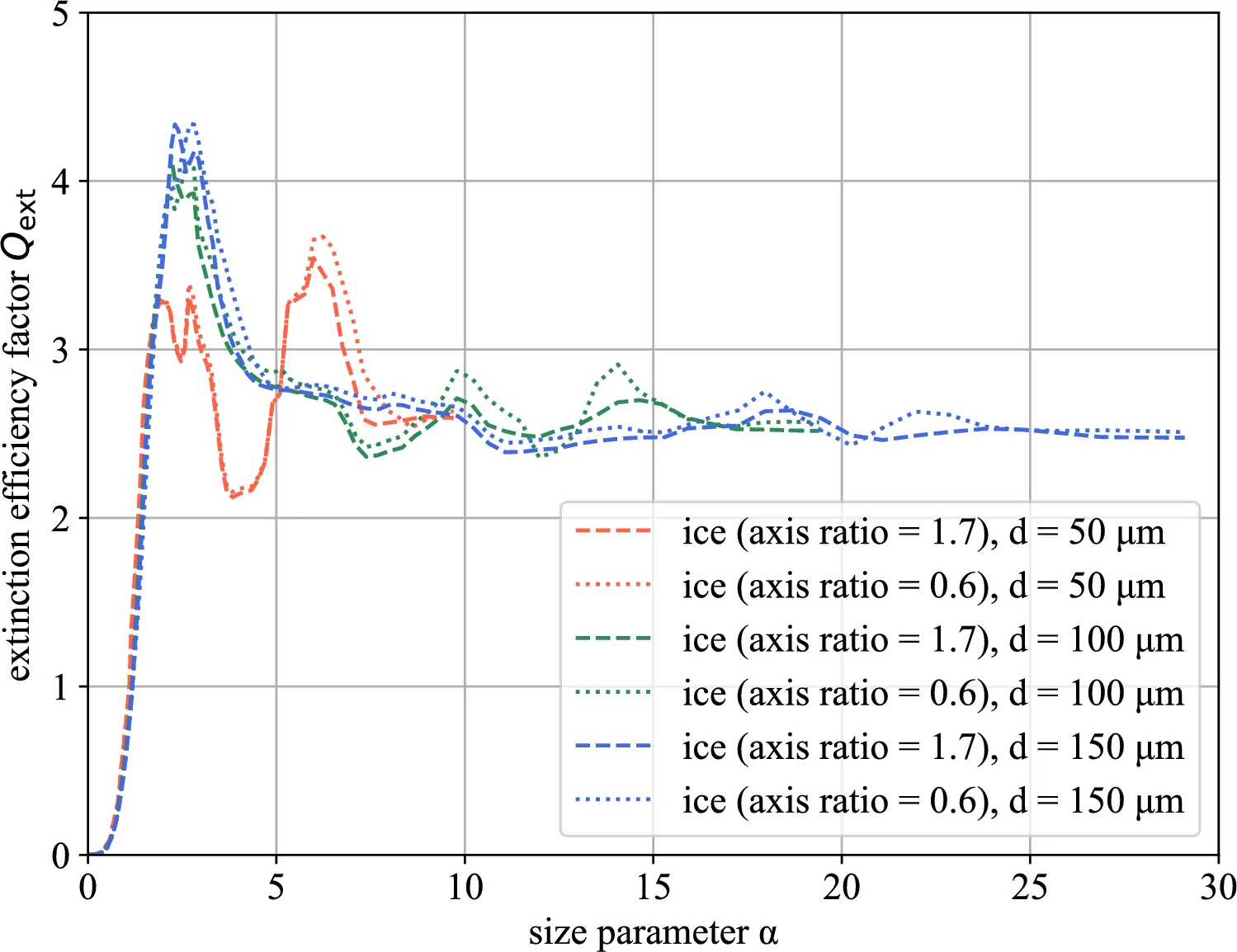
Same as Fig. 1, but for ice crystals with three different diameters and two different axis ratios.

Figure 1 shows the extinction efficiency factors for water droplets with three different diameters, plotted as a function of the size parameter *α*. The size parameter is defined as $\alpha = \pi d_\mathrm{{particle}} / \lambda$, where $d_\mathrm{{particle}}$ denotes the particle diameter and *λ* is the wavelength of the electromagnetic wave. The results indicate that the extinction efficiency factors exhibit little dependence on the diameter of water droplets. After reaching a maximum value of $Q_\mathrm{{ext}} \approx 3$ at $\alpha \approx 2$, the extinction efficiency factors decrease and asymptotically converge to a value slightly greater than 2. Figure 2 shows the extinction efficiency factors for ice crystals as a function of the size parameter, in the same manner as in Fig. 1. In this analysis, a total of six types of ice crystals were considered, corresponding to three different diameters combined with two different axis ratios. For $d_\mathrm{{particle}} = 50~\mu \textrm{m}$, the extinction efficiency factors exhibit oscillatory behavior without a pronounced maximum at a specific size parameter, gradually converging to a value slightly greater than 2. In contrast, for $d_\mathrm{{particle}} = 100$ and $150~\mu \textrm{m}$, the extinction efficiency factors reache the maximum value of $Q_\mathrm{{ext}} \approx 4$ at $\alpha \approx 2.5$, after which they oscillate and converge to a value slightly greater than 2.

From these results, it is found that for both water droplets and ice crystals, the influence on the extinction efficiency factor is limited for cloud particles with diameters larger than $d_\mathrm{{particle}} = 100~\mu \textrm{m}$. On the other hand, the extinction cross section is defined as the product of the extinction efficiency factor and the geometric cross-sectional area of a cloud particle. The PSD represents the number distribution of cloud particles as a function of their diameters. Since the geometric cross-sectional area is proportional to the square of the cloud particle diameter, the contribution of cloud particles to the extinction cross section increases with the square of their diameters. Consequently, changes in the PSD, such as an increase in cloud particle diameter, can affect the extinction cross section in a quadratic manner. Furthermore, the convergence of the extinction efficiency factor to values slightly greater than 2 in all cases is consistent with the behavior discussed in a previous study^34^.

**Table 1:** Hypothetical cloud particles used for characterizing the cloud attenuation estimation method.

Phase	Axis ratio	Diameter $d_\mathrm{{particle}}~[\mu \textrm{m}]$
Water droplets	1	50
Water droplets	1	100
Water droplets	1	150
Ice crystals	1.7	50
Ice crystals	1.7	100
Ice crystals	1.7	150
Ice crystals	0.6	50
Ice crystals	0.6	100
Ice crystals	0.6	150

### CPS sonde observations

#### Specifications and data correction

Cloud particle observation data obtained from CPS sondes and used for estimating cloud attenuation were collected in Okinawa during the Baiu (May–June) in 2016, 2017, and 2025. Because multilayered cloud systems frequently develop during the Baiu, it is important to conduct observations that specifically target such multilayered structures. Through this series of observations, CPS sondes were launched a total of ten times to vertically resolve the complex multilayered cloud structures associated with the Baiu frontal system. The CPS sonde employed in this study is a specialized cloud particle observation sonde manufactured and distributed by Meisei Electric Co., Ltd., and was released in combination with a normal radiosonde that measures basic meteorological variables such as temperature, pressure, and humidity. According to Fujiwara et al. (2016), the CPS sonde illuminates cloud particles passing through the detection volume with a laser beam and measures the scattered light (particle signals) using two detectors, thereby recording the number of cloud particles traversing the detection volume at one-second intervals. For each cloud particle signal, the voltages ($I_{55}$ and $I_\mathrm{{125p}}$), the pulse width (PW), and the degree of polarization (DOP), which is defined by equation (7) as described in Fujiwara et al. (2016), are recorded.

7 $$\begin{aligned} \textrm{DOP}=\frac{I_{55}-I_{\textrm{125p}}}{I_{55}+I_{\textrm{125p}}}. \end{aligned}$$

The DOP is bounded within the range $(-1 \le \textrm{DOP} \le 1)$. The DOP is defined as the ratio of the difference to the sum of the received signal intensities measured by the two detectors. A polarization plate is installed in front of the detector corresponding to $I_{\textrm{125p}}$, such that the received signal intensity from nearly spherical cloud particles approaches zero ($I_{\textrm{125p}} \approx 0$). In contrast, nonspherical cloud particles produce a nonzero signal intensity ($I_{\textrm{125p}} \ne 0$). Consequently, the DOP value approaches 1 for cloud particles that are nearly spherical and approaches *-1* for strongly nonspherical cloud particles. Because the CPS sonde uses laser light, nighttime operation is recommended to avoid contamination by stray sunlight. Furthermore, considering that the CPS sonde records measurements at one-second intervals, *Δ x* in equation (6) corresponds to the ascent velocity of the CPS sonde expressed in $\mathrm {m~s^{-1}}$. The observational specifications of the CPS sonde are summarized in Table 2.

The physical interpretation of these observational data is explained in the following order: correction of particle counts, interpretation of particle signals for ice crystals measured by the CPS sonde, correction of particle size, and the data averaging method. First, the correction of particle counts is applied because, when a large number of cloud particles pass through the detection volume within one second, they may be erroneously recorded as a single large particle. This correction process is therefore required to obtain accurate particle counts. Following Fujiwara et al. (2016), the correction scheme expressed by equation (8) was applied when the PW exceeded $t_\textrm{dv}$, where $t_\textrm{dv}$ denotes the time required for the CPS sonde to ascend a distance equivalent to the vertical length of the detection volume (1 cm).

8 $$\begin{aligned} \begin{aligned} N_{\textrm{cor}}&= N_{\textrm{ori}} \times 4f^3, \\ f&= \textrm{PW}/t_{\textrm{dv}}, \end{aligned} \end{aligned}$$

where $N_{\textrm{cor}}$ and $N_{\textrm{ori}}$ denote the corrected particle count and the original particle count, respectively. Next, the interpretation of particle signals for ice crystals measured by the CPS sonde requires particular care; therefore, this issue is described here. According to Fujiwara et al. (2016), a method was proposed to convert the diameters of experimental standard spherical particles into the diameters of hypothetical spherical ice particles by using scattering cross sections calculated based on Mie scattering theory. In that study, the scattering cross sections corresponding to different diameters of standard spherical particles were first calculated, and the diameters of hypothetical spherical ice particles that yielded identical scattering cross sections were then mapped to the diameters of the standard spherical particles. Subsequently, the standard spherical particles were introduced into the CPS sonde, and the corresponding values of $I_{55}$ were recorded. Through this procedure, Fujiwara et al. (2016) established a conversion from the measured $I_{55}$ values to the diameters of hypothetical spherical ice particles. Although actual ice crystals are not spherical, Fujiwara et al. (2016) argued that this approach provides a rough approximation of the equivalent volume diameters of ice crystals. In addition, it was noted that mature ice crystals may have low effective densities and porous internal structures, whereas the standard spherical particles used in the CPS experiments were solid and compact. Consequently, the equivalent volume diameters derived from this method implicitly assume ice crystals with solid internal structures. Following this interpretation, ice crystals were treated as solid particles in the calculation of scattering parameters in this study. Next, the correction of particle size is described. As noted in Fujiwara et al. (2016), the $I_{55}$ signal saturates at values exceeding approximately 7.5 V, which corresponds to cloud particle diameters of 132.87 $\mu \textrm{m}$ for water droplets and 143.49 $\mu \textrm{m}$ for ice crystals. Consequently, particularly for cloud particle diameters larger than 100 $\mu \textrm{m}$, the PSD of cloud particles derived from CPS sonde measurements differ from the actual PSD of cloud particles. Although a correction of particle size would be required for a more rigorous analysis, a methodology for such correction has not yet been well established. Therefore, no correction of particle size is applied in this study. Instead, a potential impact of this limitation on the estimated cloud attenuation is discussed in the Discussion section. Finally, the data averaging method is briefly described. The CPS sonde records cloud particle data at one-second intervals; however, within each one-second interval, it can record data for the first six cloud particles passing through the detection volume. Therefore, all cloud particles traversing the detection volume during a given one-second interval must be represented by the measurements of these first six particles. In particular, for cloud particle diameters, which are treated as equivalent volume diameters in the calculation of scattering parameters, the representative diameter for each one-second interval was defined as the cube root of the mean of the cubed diameters for the six recorded particles.

**Table 2:** Specifications of cloud particle observations obtained from CPS sondes used for cloud attenuation estimation.

Data ID	Observation date	Release time (JST)	Release location (lat., lon.)	Visually observed weather conditions	mean ascent velocity $[\mathrm {m~s^{-1}}]$
1	May 25, 2016	17:36:19	$(26^\circ 29^\prime 54^{\prime \prime }\,\textrm{N},\ 127^\circ 50^\prime 38^{\prime \prime }\,\textrm{E})$	cloudy	5.2
2	June 3, 2016	22:15:08	$(26^\circ 29^\prime 54^{\prime \prime }\,\textrm{N},\ 127^\circ 50^\prime 39^{\prime \prime }\,\textrm{E})$	light rain	5.5
3	June 7, 2016	03:05:06	$(26^\circ 29^\prime 54^{\prime \prime }\,\textrm{N},\ 127^\circ 50^\prime 39^{\prime \prime }\,\textrm{E})$	cloudy	6.6
4	June 11, 2016	20:33:25	$(26^\circ 29^\prime 54^{\prime \prime }\,\textrm{N},\ 127^\circ 50^\prime 39^{\prime \prime }\,\textrm{E})$	light rain	6.6
5	June 10, 2017	17:53:41	$(26^\circ 38^\prime 09^{\prime \prime }\,\textrm{N},\ 127^\circ 55^\prime 32^{\prime \prime }\,\textrm{E})$	cloudy	7.3
6	June 10, 2017	19:16:36	$(26^\circ 38^\prime 10^{\prime \prime }\,\textrm{N},\ 127^\circ 55^\prime 32^{\prime \prime }\,\textrm{E})$	cloudy	6.3
7	May 29, 2025	21:12:23	$(26^\circ 15^\prime 01^{\prime \prime }\,\textrm{N},\ 127^\circ 46^\prime 03^{\prime \prime }\,\textrm{E})$	cloudy	5.7
8	June 1, 2025	21:16:12	$(26^\circ 15^\prime 01^{\prime \prime }\,\textrm{N},\ 127^\circ 46^\prime 03^{\prime \prime }\,\textrm{E})$	light rain	2.8
9	June 3, 2025	21:47:42	$(26^\circ 15^\prime 01^{\prime \prime }\,\textrm{N},\ 127^\circ 46^\prime 03^{\prime \prime }\,\textrm{E})$	cloudy	4.7
10	June 4, 2025	20:59:41	$(26^\circ 15^\prime 01^{\prime \prime }\,\textrm{N},\ 127^\circ 46^\prime 03^{\prime \prime }\,\textrm{E})$	cloudy	4.6

#### Data interpretation

**Fig. 3:**
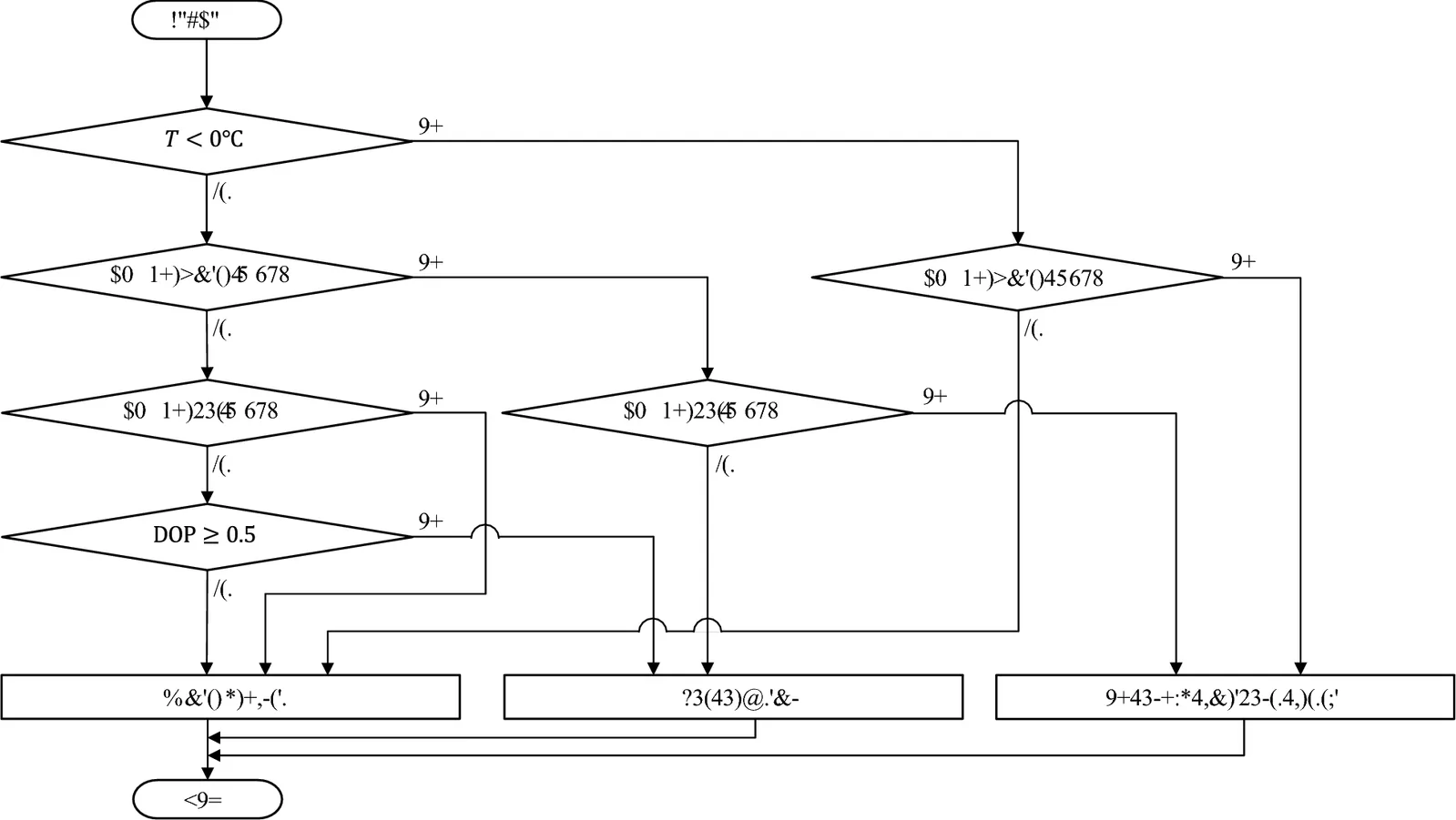
Flowchart illustrating the classification procedure used to distinguish cloud particles into water droplets and ice crystals in this study.

**Fig. 4:**
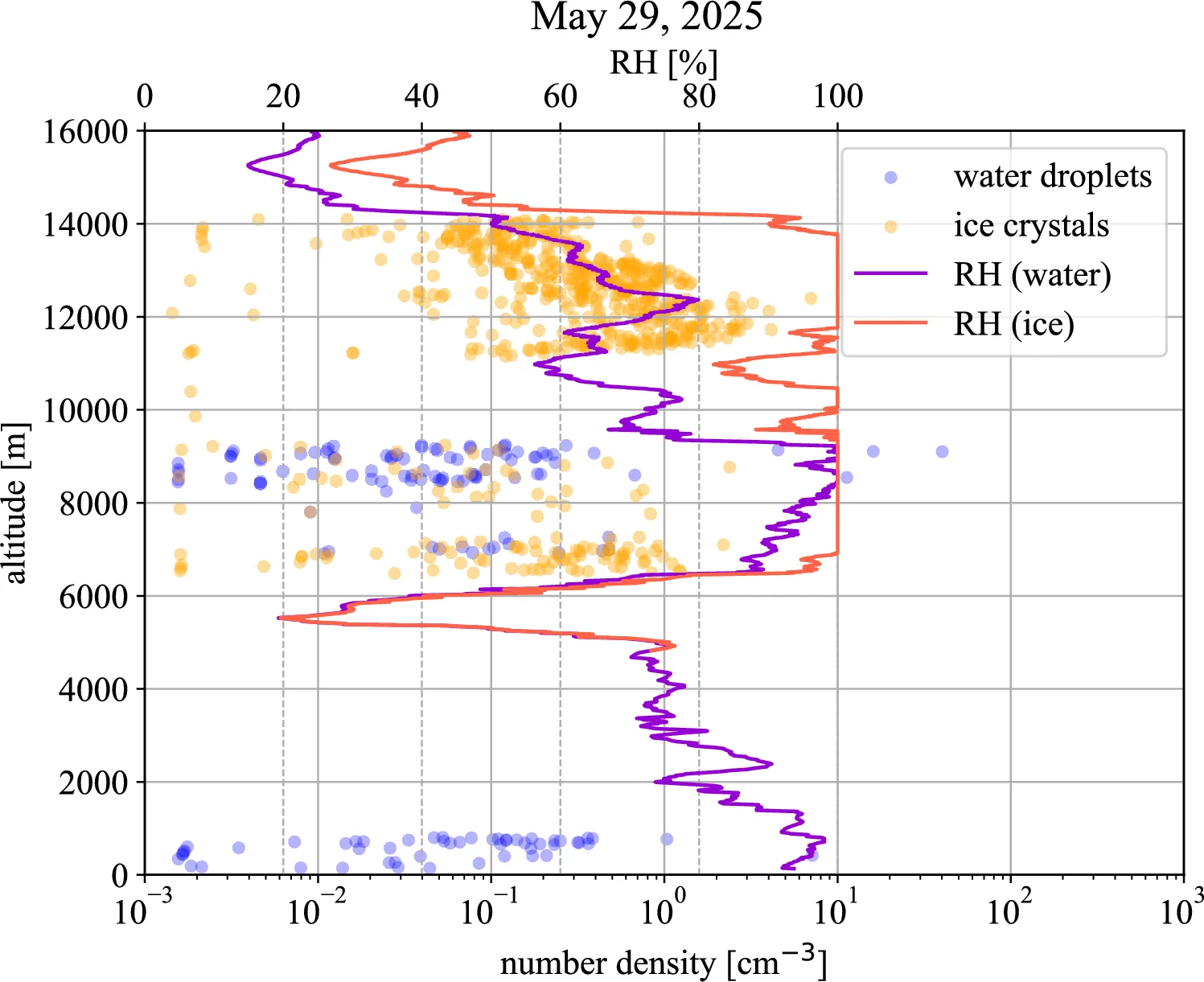
Number density profiles of cloud particles (cm$^{-3}$) observed with a CPS sonde on May 29, 2025. Cloud particles defined as water droplets are shown in blue, whereas those defined as ice crystals are shown in orange. Profiles of relative humidity with respect to water and ice are also overlaid (see the upper horizontal axis in the plot).

In this section, the interpretation of the CPS sonde observational data used in the this study is described with reference to actual measurements. As described in the Specifications and data correction section, the DOP value approaches 1 for particles that are nearly spherical and approaches *-1* for strongly non-spherical particles. Fujiwara et al. (2016) reported that particles with DOP values of 0.3 or greater tend to be water droplets, whereas those with DOP values below 0.3 tend to be ice crystals. However, a previous study that reported CPS sonde observations in the Arctic region adopted a DOP threshold of 0.5^35^. In addition, the observational results shown in Figs. 4, 7, and 10 of Fujiwara et al. (2016) suggest that a DOP threshold of 0.5 is more appropriate. In this study, our objective is to estimate cloud attenuation as realistically as possible. Therefore, we adopted a DOP threshold of 0.5 as one of the criteria for classifying cloud particles into water droplets and ice crystals. The effects of using other DOP thresholds (0.3, 0.5, and 0.7) on the estimated cloud attenuation are provided in Supplementary Figs. S1–S4 online. On the other hand, since the classification based on DOP values alone merely assumes that particles with nearly spherical shapes are water droplets and those with non-spherical shapes are ice crystals, additional constraints based on relative humidity and temperature were also considered. Specifically, water droplets were assumed to exist only when the relative humidity with respect to water exceeds 90%, whereas ice crystals were assumed to exist only when the relative humidity with respect to ice exceeds 90%. Furthermore, ice crystals were assumed not to exist in environments where the temperature is equal to or above $0^{\circ }\textrm{C}$. In summary, cloud particles in this study were classified into water droplets and ice crystals according to the following criteria. The classification procedure is also presented as a flowchart in Fig. 3.

- Water droplets were assumed to exist only when the relative humidity with respect to water exceeds 90%, whereas ice crystals were assumed to exist only when the relative humidity with respect to ice exceeds 90%. When both conditions were satisfied, the cloud was classified as a mixed-phase cloud consisting of both water droplets and ice crystals.
- Ice crystals were assumed not to exist in environments where the temperature is equal to or above $0^{\circ }\textrm{C}$.
- In mixed-phase clouds, cloud particles with a DOP value of *≥ 0.5* were defined as water droplets, whereas those with a DOP value of *< 0.5* were defined as ice crystals.

An important characteristic of the CPS sonde is that, although cloud particles can be classified into water droplets and ice crystals using parameters such as the DOP, the shape and aspect ratio of ice crystals cannot be determined. The CPS sonde fundamentally operates by detecting the signal of laser light scattered by cloud particles passing through the detection volume using two detectors with different viewing angles. However, the orientation of the cloud particles relative to the incident laser beam and the detectors is not measured. Consequently, the signals, $I_{55}$ and $I_\mathrm{{125p}}$, detected by the two detectors alone are insufficient to determine the aspect ratio of the cloud particles. Therefore, as described in the Modeling of cloud particles and T-matrix calculation settings section, representative ice crystals shapes were assumed for the analysis, namely plate-like particles with an axis ratio of 1.7 and columnar particles with an axis ratio of 0.6.

**Fig. 5:**
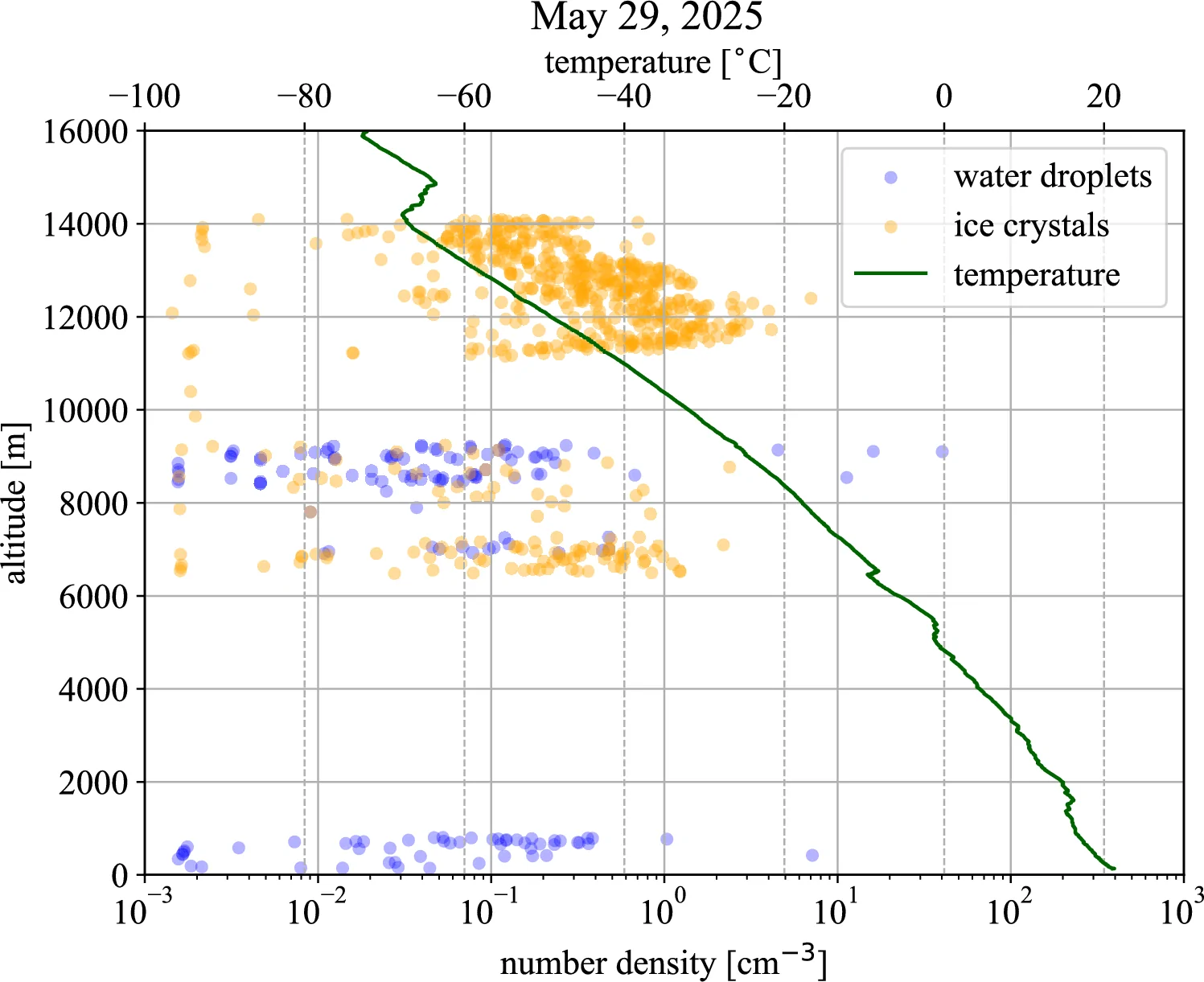
Same as Fig. 4, but with the temperature profile overlaid.

Figures 4 and 5 exemplify cloud particle data observed with a CPS sonde obtained on May 29, 2025. The relative humidity profile is overlaid in Fig. 4, while the temperature profile is overlaid in Fig. 5 for reference. For relative humidity, two quantities are presented: the observed relative humidity with respect to water and the calculated relative humidity with respect to ice. Values of relative humidity with respect to ice that exceeded 100% were plotted as 100%. In both figures, cloud particles defined as water droplets are indicated in blue, and those defined as ice crystals are indicated in orange. Since the CPS sonde records observational data only for the first six cloud particles detected within each second, the plotted values represent the product of the corrected particle count and the fraction of water droplets in blue, and the product of the corrected particle count and the fraction of ice crystals in orange. This cloud particle data indicates the presence of four distinct cloud layers. Cloud layers composed of water droplets were observed below an altitude of 1 km and in the altitude range of 8–10 km. In contrast, cloud layers composed of ice crystals were identified in the altitude ranges of 6–8 km and 11–14 km. Focusing on the temperature in each cloud layer, it was above $0^\circ \textrm{C}$ below an altitude of 1 km, between $0^\circ \textrm{C}$ and $-40^\circ \textrm{C}$ in the altitude range of 6–10 km, and below $-40^\circ \textrm{C}$ in the altitude range of 11–14 km. These findings are consistent with the meteorological constraint that supercooled water droplets can exist within the temperature range between $0^\circ \textrm{C}$ and $-40^\circ \textrm{C}$, whereas only ice crystals can exist at temperatures below $-40^\circ \textrm{C}$^17^.

#### Data classification

To check the dependency on weather condition, the cloud particle data obtained from CPS sondes (Table 2) were classified into two groups according to the following criteria. Group 1 (G1) includes observations for which, based on archived weather charts provided by the Japan Meteorological Agency, the observation site was located near the front or the weather was light rain. Group 2 (G2) consists of all remaining cases not categorized as G1. As a result of this classification, the cloud particle data were grouped as summarized in Table 3.

**Table 3:** Classification of cloud particle data obtained from CPS sondes.

Group	Data ID numbers presented in Table 2
G1	2, 3, 4, 8
G2	1, 5, 6, 7, 9, 10

## Results

**Fig. 6:**
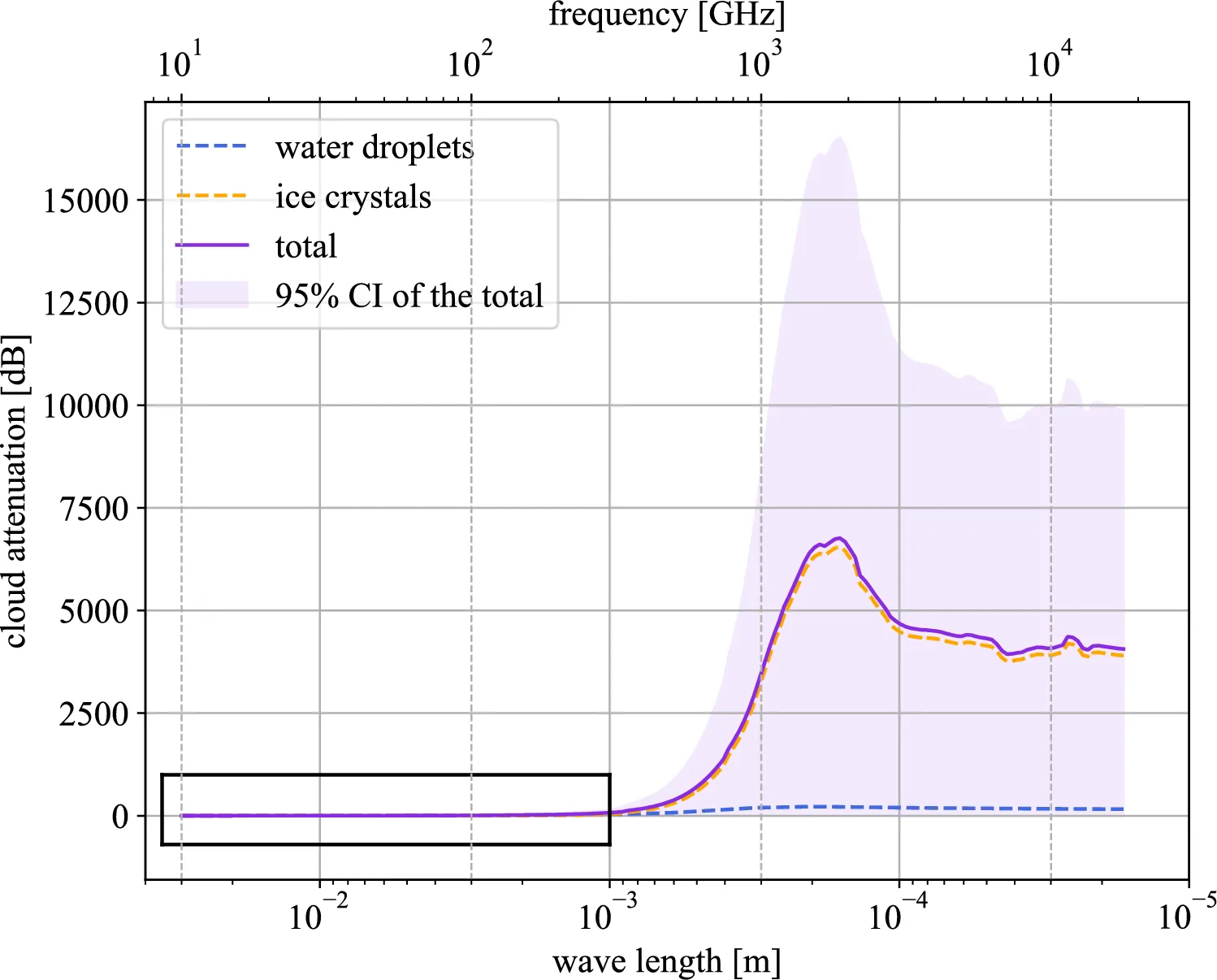
Mean cloud attenuation of G1 as a function of wavelength, covering the range from 3.0 cm to approximately $20~\mu \textrm{m}$. The blue and orange dashed lines represent the mean cloud attenuation due to water droplets and ice crystals, respectively, while the solid blue line indicates the mean total cloud attenuation. The lightly blue hatched area denotes the 95% CI of the mean total cloud attenuation. The black rectangle highlights the wavelength range shown as a close-up in Fig. 7.

**Fig. 7:**
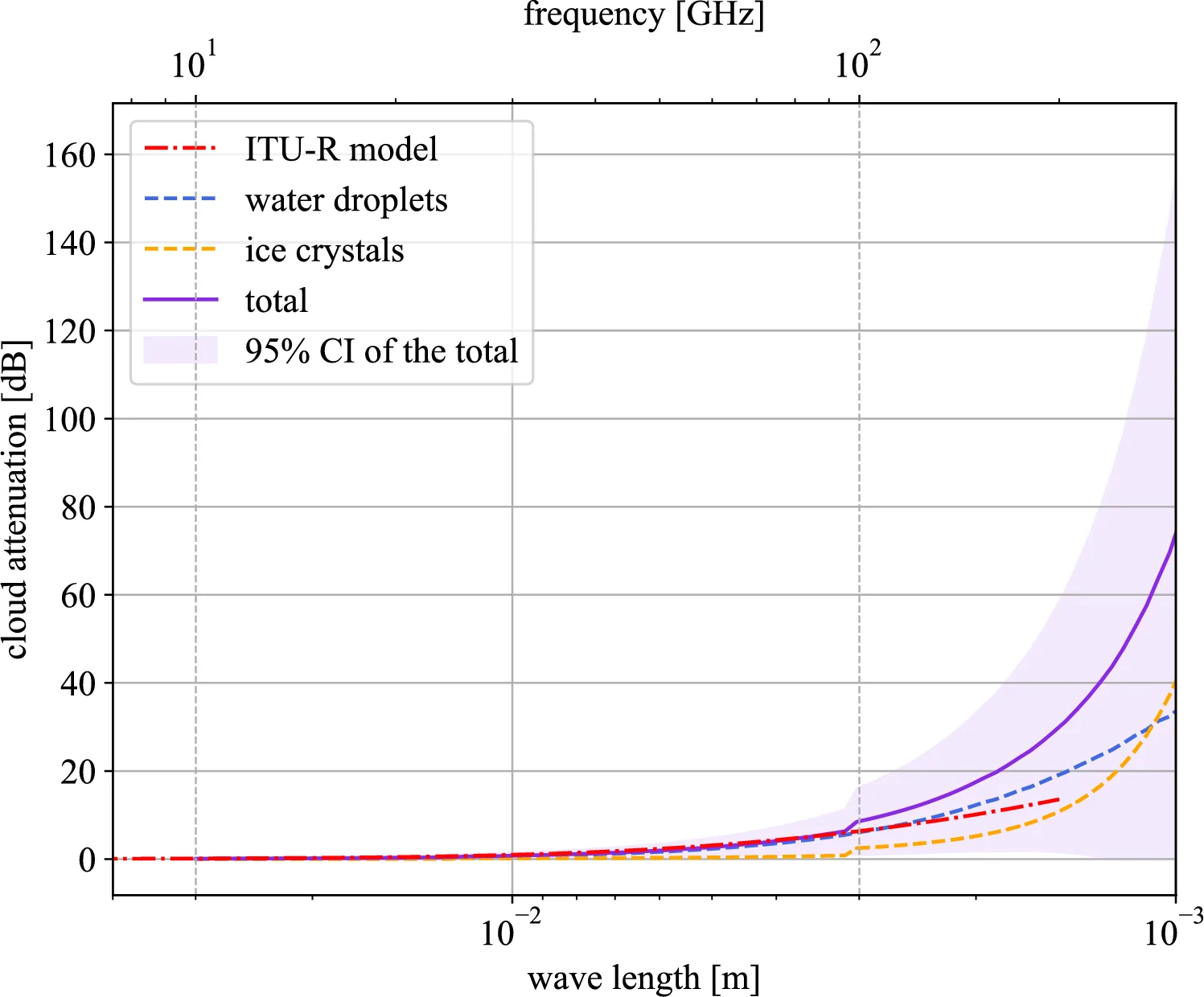
Same as Fig. 6, but covering the wavelength range from 3.0 cm to 1.0 mm, with the cloud attenuation predicted by the ITU-R model indicated by a red dash-dotted line.

Figures 6 and 7 show the mean cloud attenuation of G1 as a function of wavelength. Figure 6 covers wavelengths from 3.0 cm to approximately $20~\mu \textrm{m}$ (10 GHz–10 THz), and Fig. 7 shows the subset bounded by the black rectangle in Fig. 6, corresponding to wavelengths from 3.0 cm to 1.0 mm (10 GHz–300 GHz). In the figures, the orange and blue dashed lines represent the mean cloud attenuation due to ice crystals and water droplets, respectively, and the blue solid line indicates the mean total cloud attenuation. In addition, Fig. 7 presents, for comparison, the cloud attenuation predicted by the ITU-R model over the frequency range up to 200 GHz (approximately 1.5 mm in wavelength), shown as a red dash-dotted line. The cloud attenuation predicted by the ITU-R model was derived using the LWP calculated for all water droplets from their equivalent volume diameters, the cloud layer thickness, and the density of liquid water. In addition, the lightly blue hatched area denotes the 95% confidence interval (95% CI) of the mean total cloud attenuation. To account for the small sample size, the 95% CI was estimated based on the t-distribution and an unbiased estimate of variance. Because cloud attenuation cannot take negative values, portions of the 95% CI that fall below zero are not shown. The mean total cloud attenuation reached approximately 6,800 dB at a peak near a wavelength of about $200~\mu \textrm{m}$. At wavelengths shorter than the peak, the mean total cloud attenuation decreased and asymptotically approached about 4,000 dB. Such extreme cloud attenuation implies that the energy of electromagnetic waves incident on the cloud is almost entirely lost due to absorption or scattering. In other words, an incident photon is nearly certain to experience absorption or scattering within the cloud. From the perspective of satellite communications, this indicates that communication systems based on sub-THz, THz, and FSO links are not feasible under such conditions. On the other hand, in conventional satellite link design, previous studies have reported that attenuation margins of roughly 10 dB are required to accommodate rain and cloud attenuation^36^. Therefore, a reliable communication is feasible at wavelengths longer than approximately 3 mm (corresponding to about 100 GHz). Focusing on the frequency range below 200 GHz, the cloud attenuation predicted by the ITU-R model shows values comparable to those due to water droplets down to a wavelength of approximately 2.5 mm. However, at wavelengths shorter than approximately 2.5 mm, the ITU-R model slightly underestimates the cloud attenuation. This provides strong evidence that the ITU-R model considers only liquid water. Even at frequencies below 200 GHz, the values predicted by the ITU-R model fall below those indicated by the blue solid line, which represents the total cloud attenuation including the effects of ice crystals. As another notable feature, at wavelengths longer than 1.0 mm, the contribution from water droplets to cloud attenuation was larger, whereas at wavelengths shorter than 1.0 mm cloud attenuation was dominated by ice crystals. Furthermore, examination of the 95% CI revealed that the maximum cloud attenuation approached over 15,000 dB, indicating a large degree of variability.

**Fig. 8:**
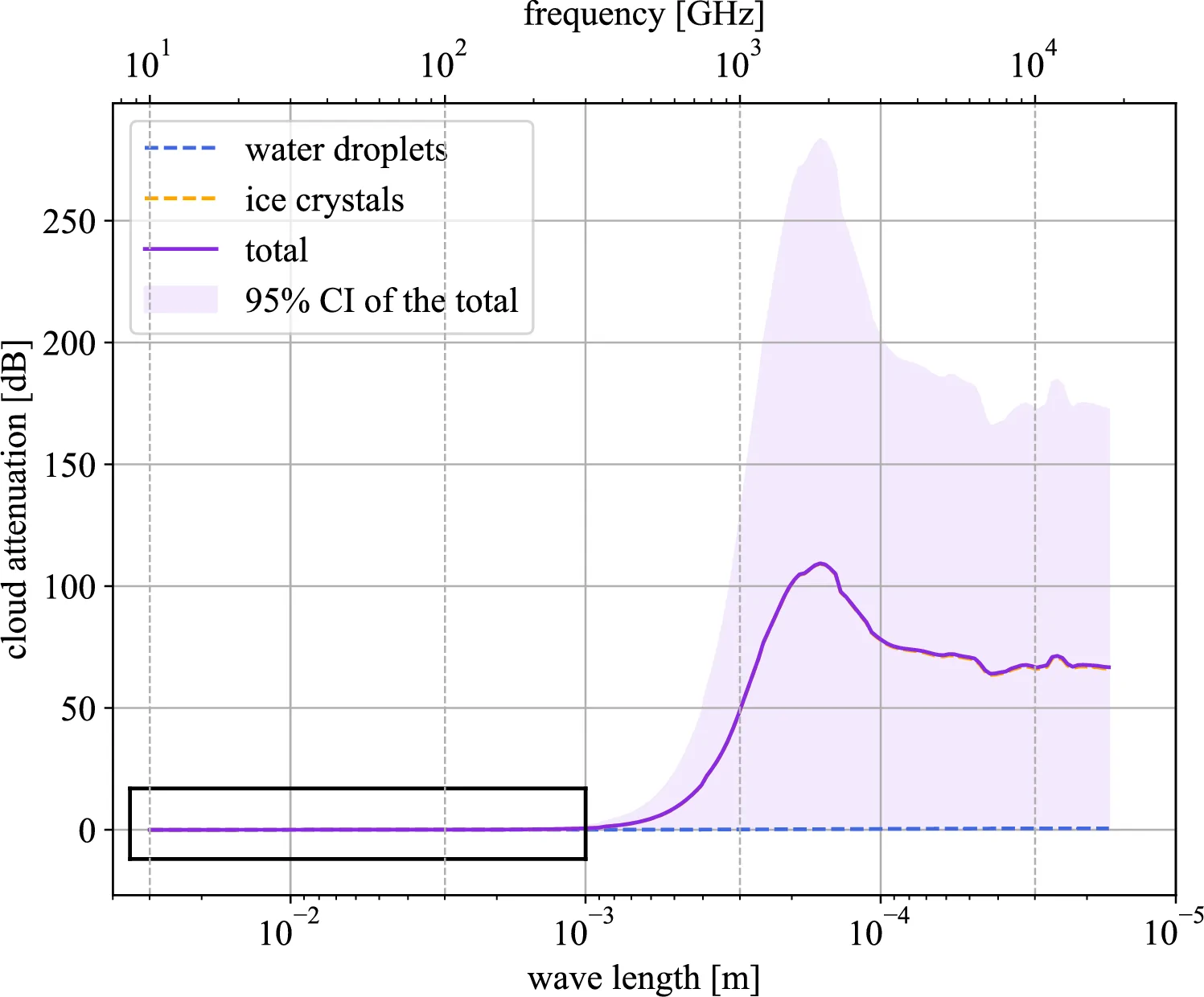
Mean cloud attenuation of G2 as a function of wavelength, covering the range from 3.0 cm to approximately $20~\mu \textrm{m}$. The blue and orange dashed lines represent the mean cloud attenuation due to water droplets and ice crystals, respectively, while the solid blue line indicates the mean total cloud attenuation. The lightly blue hatched area denotes the 95% CI of the mean total cloud attenuation. The black rectangle highlights the wavelength range shown as a close-up in Fig. 9.

**Fig. 9:**
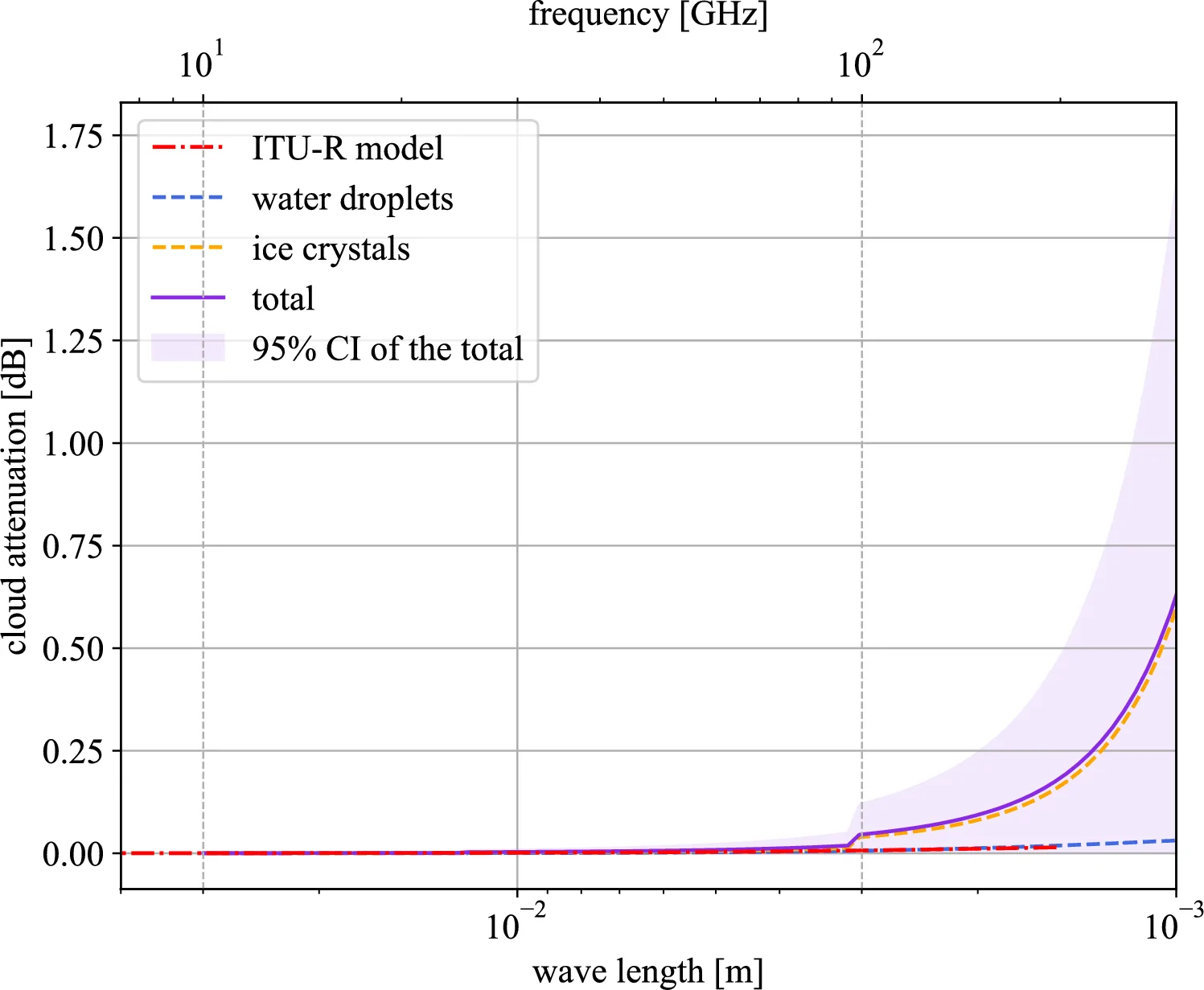
Same as Fig. 8, but covering the wavelength range from 3.0 cm to 1.0 mm, with the cloud attenuation predicted by the ITU-R model indicated by a red dash-dotted line.

Figures 8 and 9 show the mean cloud attenuation of G2 as a function of wavelength. The wavelength ranges shown are the same as those in Figs. 6 and 7: Figure 8 covers wavelengths from 3.0 cm to approximately $20~\mu \textrm{m}$ (10 GHz–10 THz), and Fig. 9 covers wavelengths from 3.0 cm to 1.0 mm (10 GHz–300 GHz). The line styles and colors in both figures, as well as the lightly blue hatched area, represent the same quantities as in Figs. 6 and 7, and the black rectangle in Fig. 8 denotes the close-up region shown in Fig. 9. For G2, the mean total cloud attenuation reached a peak value of approximately 110 dB at a wavelength near $200~\mu \textrm{m}$. At wavelengths shorter than the peak, the mean total cloud attenuation decreased and asymptotically approached about 70 dB. Conversely, at wavelengths longer than approximately $500~\mu \textrm{m}$, the mean total cloud attenuation fell below 10 dB, corresponding to typical rain and cloud attenuation margins for satellite communications. This suggests that satellite communication is feasible at wavelengths longer than approximately $500~\mu \textrm{m}$ (corresponding to about 600 GHz) under cloud conditions classified as G2, indicating a potential viability of sub-THz communications in such environments. It should be noted that this interpretation is conducted under the condition that the estimated cloud attenuation represents a lower bound, because the PSD of cloud particles may have been underestimated due to the detection limits of the CPS sondes, as described in the Specifications and data correction section. Further details are provided in the Discussion section. On the other hand, with regard to THz and FSO, it was suggested that even under cloud conditions classified as G2, satellite communication would remain difficult. Focusing on the cloud attenuation predicted by the ITU-R model in the frequency range below 200 GHz, it shows values comparable to those due to water droplets, similar to the case of G1. However, unlike in the case of G1, there was no noticeable deviation from the cloud attenuation due to water droplets. In the case of G2, cloud attenuation due to water droplets was nearly zero, and most of the cloud attenuation was attributable to ice crystals; therefore, the ITU-R model substantially underestimated the total cloud attenuation even at frequencies below 200 GHz. Similar to G1, cloud attenuation due to ice crystals was dominant at wavelengths shorter than 1.0 mm, whereas in G2 it remained dominant even at wavelengths longer than 1.0 mm.

**Fig. 10:**
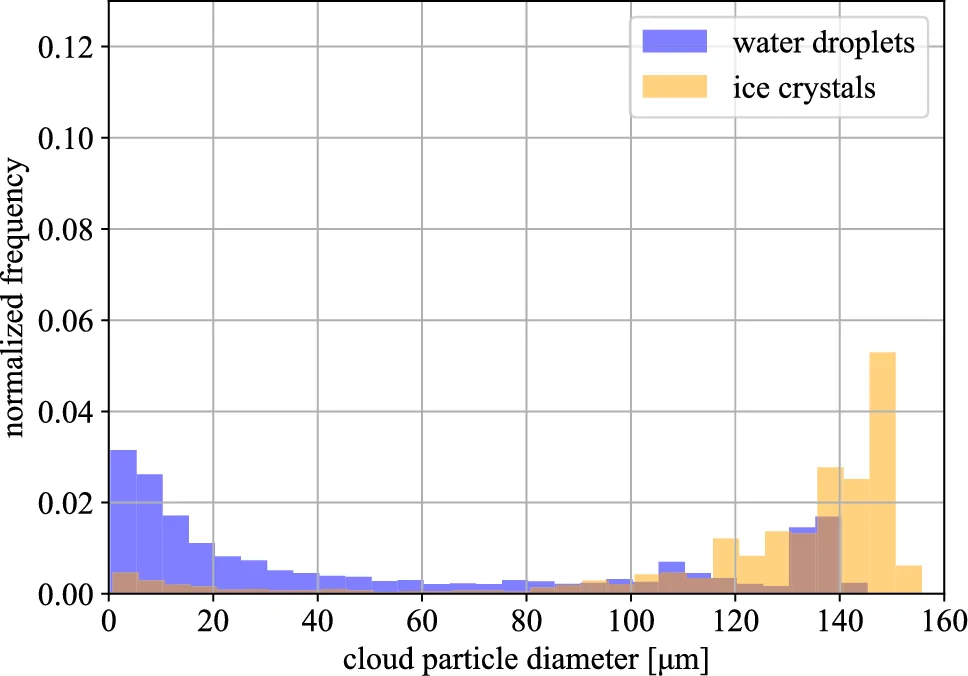
PSD of water droplets and ice crystals for G1. The horizontal axis shows cloud particle diameter, and the vertical axis indicates the normalized frequency. All data classified as G1 are included. The histogram is constructed from representative particle diameters derived from CPS sonde measurements taken at one-second intervals.

**Fig. 11:**
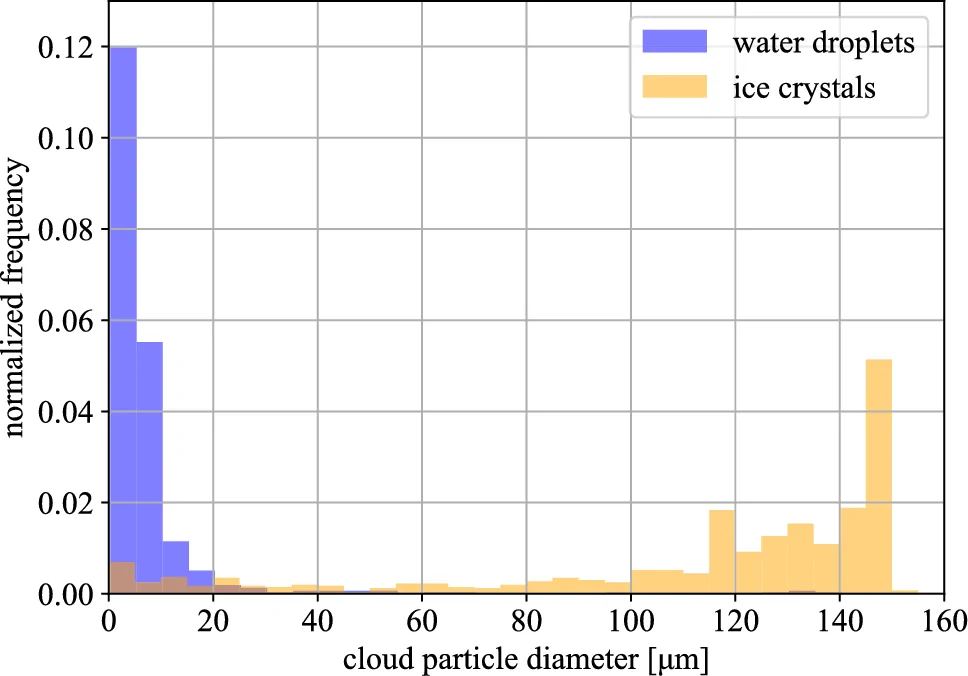
Same as Fig. 10, but for G2.

Figures 10 and 11 show the PSD of water droplets and ice crystals for G1 and G2, respectively. The PSD presented here were constructed as histograms of representative particle diameters derived from CPS sonde measurements taken at one-second intervals. Specifically, six measured particle diameters were classified into water droplets and ice crystals based on DOP, and for each class the representative diameter was defined as the cube root of the mean of the cubed diameters. Subsequently, water droplets and ice crystals to be excluded were filtered out based on the relative humidity and temperature conditions. All data belonging to G1 and G2 were accumulated, and the vertical axis of each histogram was normalized separately for water droplets and ice crystals. For ice crystals, both G1 and G2 exhibited a peak near $150~\mu \textrm{m}$; however, this feature arises from the detection limits of CPS sondes, as described in the Specifications and data correction section. Therefore, Figs. 10 and 11 do not accurately represent the actual PSD of ice crystals. In contrast, the distributions of water droplets differed between G1 and G2. In G1, peaks were observed in the region below $20~\mu \textrm{m}$ and near $135~\mu \textrm{m}$. In G2, a peak appeared below $20~\mu \textrm{m}$, whereas no corresponding peak near $135~\mu \textrm{m}$ was observed. The common peak below $20~\mu \textrm{m}$ indicates that relatively small cloud particles are primarily composed of water droplets, suggesting that water droplets tend to be smaller than ice crystals when considering the overall cloud particle population. When the phase of cloud particles is not considered, such phase-dependent tendencies in cloud particle size can be overlooked. This is particularly problematic in regimes where the cloud particle size is no longer negligible relative to the wavelength, leading to inaccuracies in the estimation of cloud attenuation. Even at frequencies below 200 GHz, the ITU-R model represents cloud particle properties solely by parameters such as LWC and LWP, which results in discrepancies in the estimated cloud attenuation. Consequently, the phase-dependent characteristics of cloud particle size constitute an important consideration for satellite communications. The peak near $135~\mu \textrm{m}$ observed in G1 is also attributable to the detection limits of CPS sondes, similar to the case for ice crystals. Such comparatively large particles may represent drizzle or small raindrops formed through collision–coalescence growth of water droplets.

## Discussion

A salient feature of the estimated cloud attenuation obtained in this study (Figs. 6–9) is that, particularly at wavelengths shorter than 1.0 mm, cloud attenuation due to ice crystals becomes dominant. In previous studies on cloud attenuation in satellite communications, it has been argued that attenuation due to water droplets is dominant, whereas attenuation due to ice crystals has been neglected^12,13^ or treated as a small constant^14,15^. In emerging satellite communications based on sub-THz, THz, and FSO, which are expected to expand in the coming years, cloud attenuation due to ice crystals can no longer be neglected and must be addressed with careful consideration. Conventional cloud attenuation models are no longer sufficient to provide reliable estimates for satellite communications operating in the sub-THz, THz, and FSO regimes. The results presented in this study provide a strong foundation for such efforts. In particular, with respect to the PSD of cloud particles, as shown in Figs. 10 and 11, ice crystals tend to exhibit larger diameters than water droplets. These phase-dependent tendencies in cloud particle size constitute a crucial parameter in discussing cloud attenuation due to ice crystals, and this perspective has been revealed precisely because in situ observational data were employed. To the best of our knowledge, this study is the first to highlight the importance of ice crystals in cloud attenuation for satellite communications, based on in situ cloud particle observations obtained using CPS sondes.

It should be noted that, as described in the Specifications and data correction section, the PSD of cloud particles obtained from CPS sondes do not accurately represent the actual PSDs for cloud particles larger than $100~\mu \textrm{m}$ due to the detection limits of the instrument. Therefore, the estimated cloud attenuation in this study, particularly that attributed to ice crystals, should be regarded as a potential lower bound. As discussed in the Characteristics of the cloud attenuation estimation method section, cloud attenuation is expected to increase in proportion to the squared actual PSD of cloud particles, which suggests that the true cloud attenuation may be larger than the values estimated here. From this perspective as well, the assertion that ice crystals can become dominant in cloud attenuation remains robust.

Here, we discuss another possible reason why cloud attenuation due to ice crystals becomes dominant in the short-wavelength region. At a wavelength of 3.0 cm, the order of magnitude of the imaginary part of the complex refractive index is approximately $10^0$ for water and $10^{-4}$ for ice. In contrast, at a wavelength of $20~\mu \textrm{m}$, the imaginary parts for both water and ice are on the order of $10^{-1}$. This indicates that, as the wavelength decreases, the imaginary part of the complex refractive index of ice becomes relatively larger, leading to a stronger absorption contribution from ice crystals. As a result, cloud attenuation due to ice crystals is expected to become dominant in the short-wavelength region, such as wavelengths on the order of micrometers. Therefore, the cloud attenuation associated with ice crystals may have been underestimated in previous studies. This finding highlights the necessity of re-evaluating ice-crystal-induced cloud attenuation, particularly for satellite communication systems employing sub-THz, THz, and FSO.

Next, we focus on the differences in cloud attenuation between the G1 and G2 classifications. G1 corresponds to cases in which the observation site was located along the Baiu front or the weather conditions were light rain, and the cloud attenuation estimated for G1 was orders of magnitude larger than that for G2. For those cases within G1 that were recorded as light rain, the precipitation rates in the vicinity of the sonde release time were on the order of several millimeters per hour, based on data from the Japan Meteorological Agency. Therefore, while rain attenuation may be present, it is unlikely to be orders of magnitude larger than cloud attenuation. In order to make the most effective use of the valuable observational data acquired during rain-cloud events, the light-rain cases were also incorporated into the analysis. As is generally known, frontal zones are characterized by the intrusion of cold air beneath warm air, which lifts the warm air along the frontal surface. This process induces updrafts and creates favorable conditions for water vapor condensation. In particular, during the Baiu, it has been reported that lower tropospheric southerly winds transport water vapor from the ocean into the Baiu front, and wind convergence induces large-scale vertical circulation, leading to the development of intense precipitating cloud systems^37,38^. Therefore, the larger cloud attenuation in G1 compared with G2 is consistent with these meteorological characteristics. Furthermore, the magnitude of the cloud attenuation estimated for G1 is in agreement with the order of cloud attenuation reported in the previous study for nimbostratus and cumulonimbus clouds^14^. Since no substantial discrepancy is observed, the cloud attenuation estimates obtained in this study are considered to be reasonable.

## Conclusions

A key contribution of this study is the estimation of cloud attenuation based on in situ cloud particle observations, which highlights the limitations of conventional cloud attenuation models in satellite communications. In situ observations of cloud particles were conducted using CPS sondes in the vicinity of the Baiu front during May–June 2016, 2017, and 2025. The estimation was conducted based on Mie scattering theory in conjunction with the radiative transfer equation, considering both liquid and ice phases which is different from conventional cloud attenuation models. The observations were classified into two groups according to the weather condition on each observation day and evaluated separately.

The results showed that when the observation site was located along the Baiu front or when weather conditions were light rain, a reliable satellite communication is feasible at wavelengths longer than approximately 3 mm (corresponding to about 100 GHz). On the other hand, when the observation site was neither located along the Baiu front nor experiencing rainfall, a reliable satellite communication is feasible at wavelengths longer than approximately $500~\mu \textrm{m}$ (corresponding to about 600 GHz). Cloud attenuation due to ice crystals becomes dominant particularly at wavelengths shorter than 1.0 mm. This behavior is considered to be attributable to the tendency for ice crystals to have larger particle sizes than water droplets, as well as to the relative increase in the magnitude of the imaginary part of the refractive index of ice with decreasing wavelength. Furthermore, because previous studies have evaluated ice clouds primarily in terms of thin cirrus, the contribution of ice crystals to cloud attenuation may have been underestimated. The present results highlight the importance of reassessing the impact of ice crystals when estimating cloud attenuation, given that conventional cloud attenuation models, including the ITU-R model, either neglect their effects or treat them as a small constant.

In this study, we examined the limitations of the ITU-R model for frequencies below 200 GHz and additionally investigated cloud attenuation including the effects of ice crystals down to wavelengths of approximately $20~\mu \textrm{m}$ (corresponding to about 10 THz). Although this represents an initial validation in a specific temporal and geographical context (Okinawa during the Baiu), similar investigations conducted worldwide could contribute to improving the globally applicable and widely used ITU-R model into a more useful framework for broader research and engineering communities. To achieve this, similar observations considering seasonal variability and geographical dependence are required.

## Supplementary Information

Supplementary Information.

## Data Availability

The CPS sonde observational data supporting the conclusions of this study are available from the corresponding author upon reasonable request. Requests for the data should be addressed to the authors.
