# Supplementary material for: Cloud attenuation estimation based on cloud particle sensor sonde observations for next-generation satellite communications
Source: Sci Rep. 2026 Jul 8;16:27780. doi: 10.1038/s41598-026-61143-z (PMC13545205; doi:10.1038/s41598-026-61143-z)
Supplement: Supplementary file 1 [file 41598_2026_61143_MOESM1_ESM.pdf]

Supplementary Figures

for

Cloud Attenuation Estimation Based on Cloud Particle Sensor Sonde Observations for Next-Generation Satellite Communications

Takahiro Ohno[1,2], Munehiro Matsui[1], Kiyohiko Itokawa[1], Tomohiro Tokuyasu[1], Kosuke Ito[3], Shoichi Shige[2], Tetsuya Takemi[3]

[1]Access Network Service Systems Laboratories, NTT, Inc., Yokosuka, 239-0847, Japan
[2]Graduate School of Science, Kyoto University, Kyoto, 606-8502, Japan
[3]Disaster Prevention Research Institute, Kyoto University, Uji, 611-0011, Japan

Supplementary Notes

These supplementary figures show the variation in cloud attenuation estimates resulting from changes in the threshold of the DOP, which is used to classify cloud particles observed by the CPS sondes into water droplets and ice crystals. The influence of the DOP threshold on the estimated cloud attenuation is examined. Here, we present the cloud attenuation obtained when the DOP threshold is set to 0.3, 0.5, and 0.7.

Supplementary Figure S1. Mean cloud attenuation for different DOP thresholds in G1

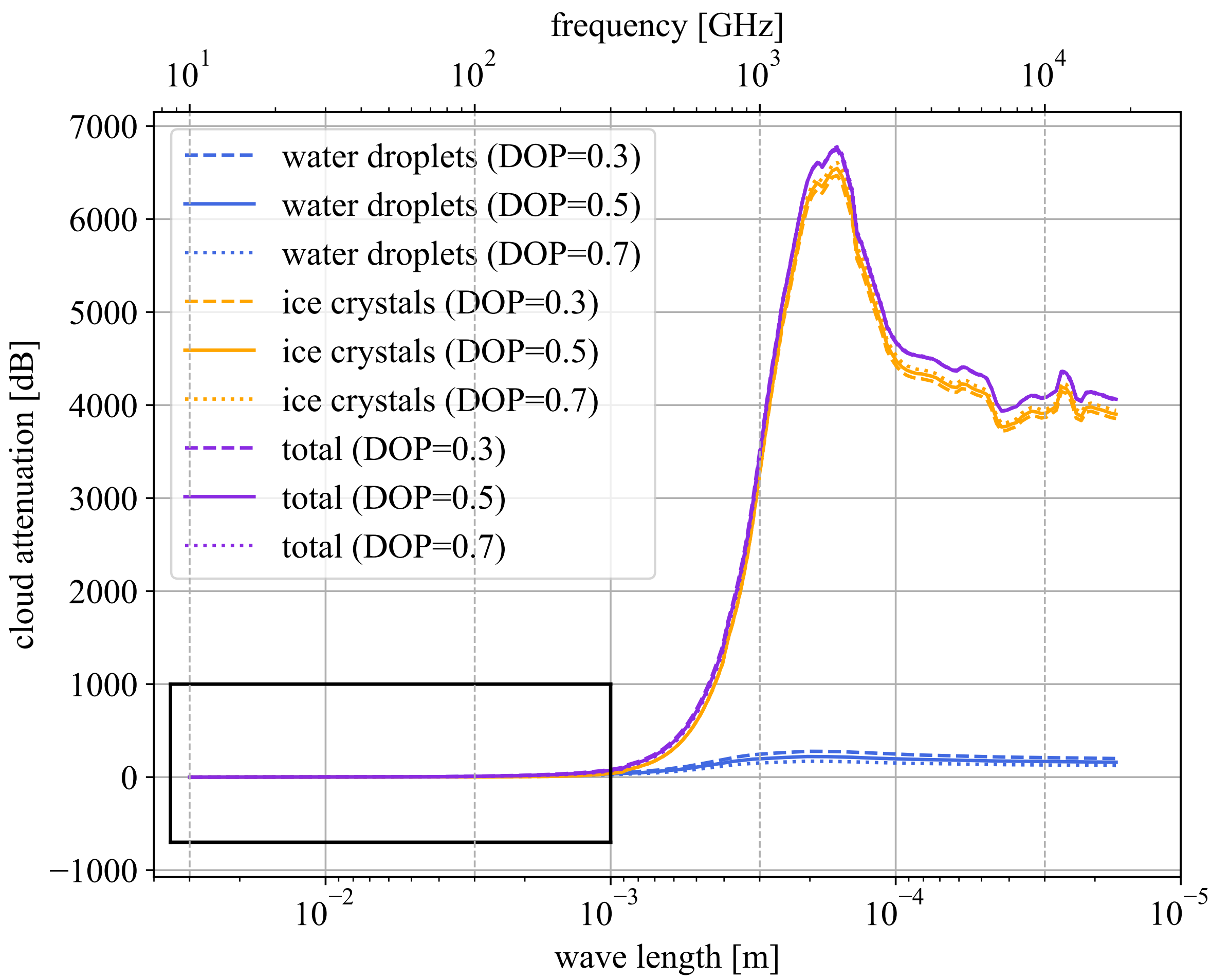

Supplementary Figure S2. Mean cloud attenuation for different DOP thresholds in G1 (zoomed view)

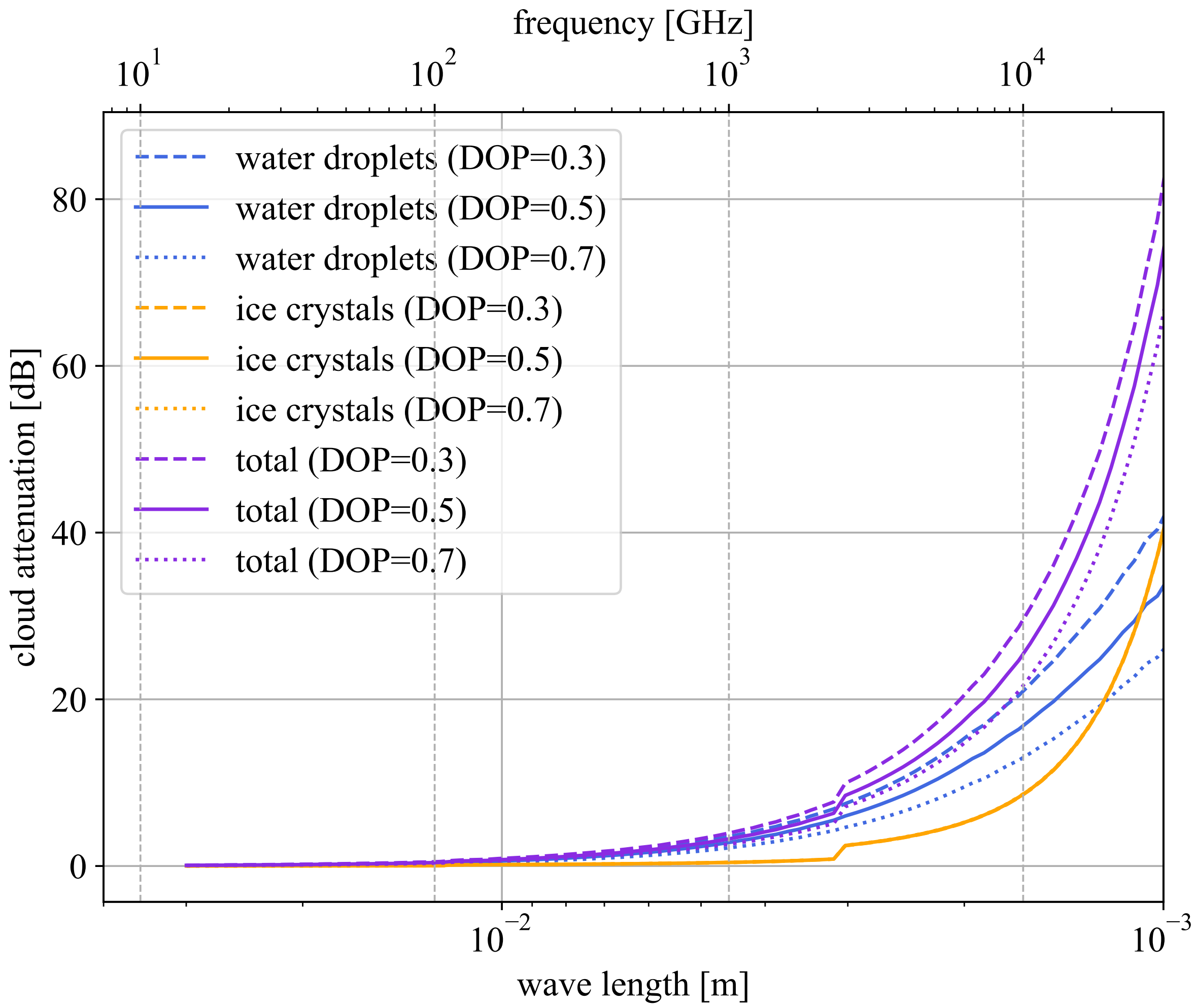

Supplementary Figure S3. Mean cloud attenuation for different DOP thresholds in G2

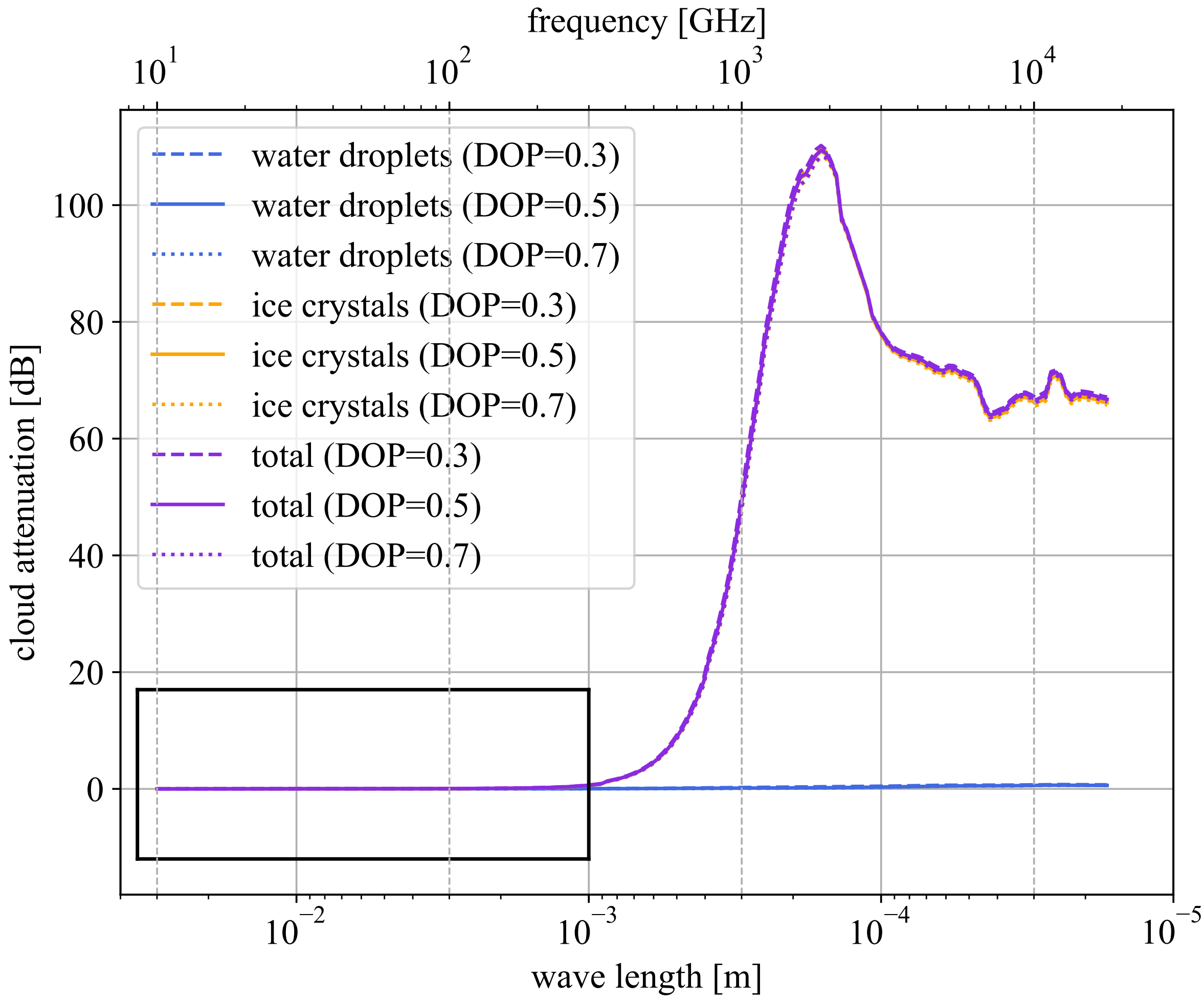

Supplementary Figure S4. Mean cloud attenuation for different DOP thresholds in G2 (zoomed view)

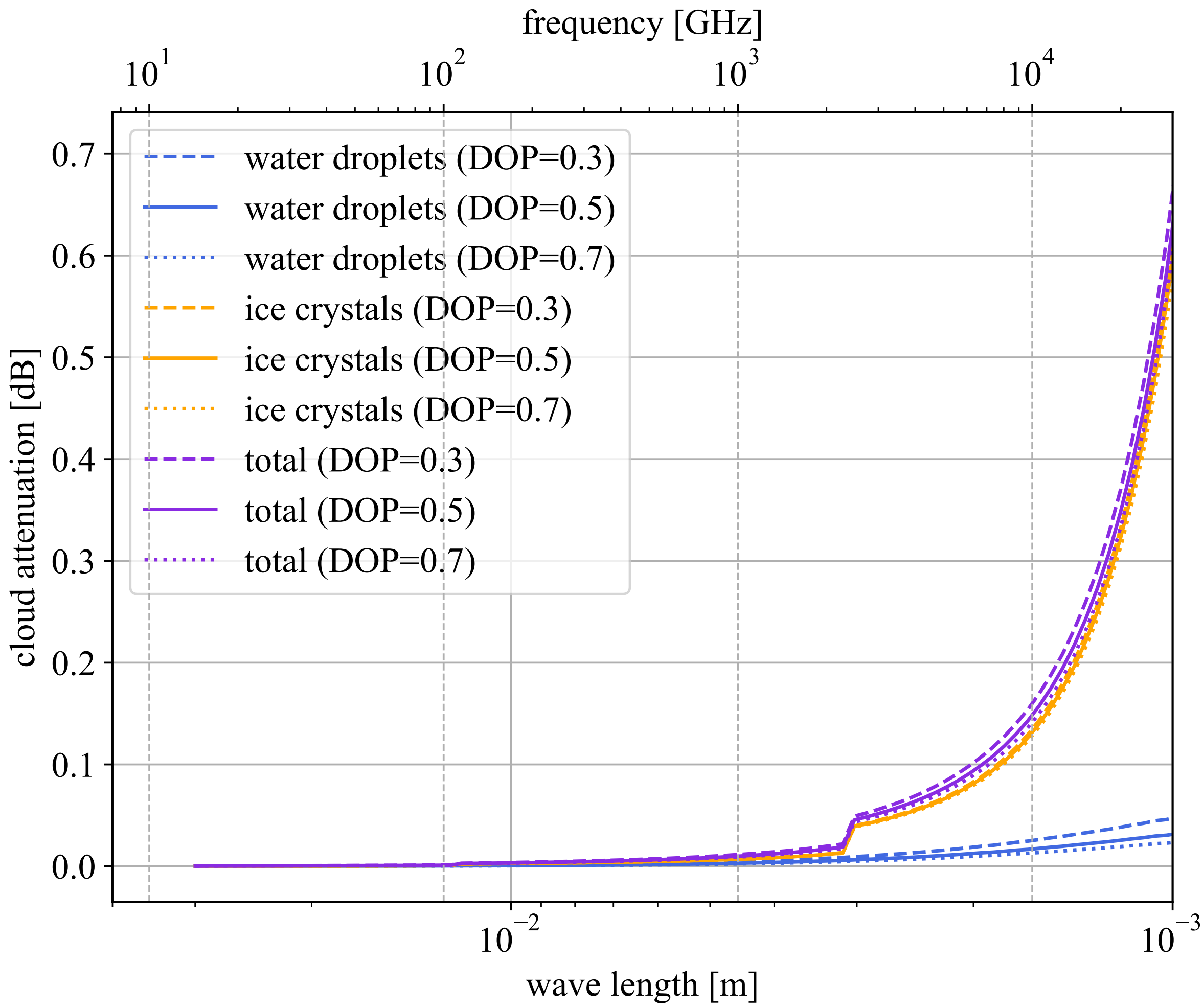

Discussion

The DOP takes values between -1 and 1 depending on the shape of detected cloud particles. As described in the main text, the DOP value approaches 1 for spherical particles and approaches -1 for nonspherical particles. In this study, nonspherical particles are regarded as ice crystals; therefore, a higher DOP threshold leads to a greater likelihood of classifying cloud particles as ice crystals.

As shown in Figs. S1 and S3, the total cloud attenuation for both G1 and G2 exhibits little dependence on the DOP threshold. In contrast, for G1, differences associated with the DOP threshold are observed when considering the "water droplets" and "ice crystals" components separately. For the "water droplets" case, the cloud attenuation is larger when the DOP threshold is 0.3, whereas for the "ice crystals" case, it is larger when the DOP threshold is 0.7. This behavior is consistent with the fact that a higher DOP threshold increases the likelihood of classifying cloud particles as ice crystals. From the enlarged views in Figs. S2 and S4 over the wavelength range from 3 cm to 1 mm, the cloud attenuation for the "ice crystals" case shows little dependence on the DOP threshold. In contrast, the "water droplets" case exhibits some differences depending on the DOP threshold, with larger attenuation estimated at a threshold of 0.3 for both G1 and G2. In particular, for G1, the difference exceeds 10 dB depending on the chosen DOP threshold.

From the above analysis, no significant differences in cloud attenuation were observed across DOP thresholds when considered overall. However, for cases such as G1, where well-developed clouds associated with the baiu front are present, a more precise evaluation of cloud attenuation requires careful selection of an appropriate DOP threshold, taking into account the characteristics of the CPS sonde in relation to cloud particle shape. To achieve this, it is necessary to compare CPS sonde observations with measurements from instruments capable of directly imaging cloud particles, such as video sondes. Simultaneous observations using both video sondes and CPS sondes therefore remain an important subject for future work.
